# Sublethal effects of imidacloprid on targeting muscle and ribosomal protein related genes in the honey bee *Apis mellifera* L.

**DOI:** 10.1038/s41598-017-16245-0

**Published:** 2017-11-21

**Authors:** Yan-Yan Wu, Qi-Hua Luo, Chun-Sheng Hou, Qiang Wang, Ping-Li Dai, Jing Gao, Yong-Jun Liu, Qing-Yun Diao

**Affiliations:** 1 Key Laboratory of Pollinating Insect Biology, Ministry of Agriculture, Institute of Apicultural Research, Chinese Academy of Agricultural Sciences, Beijing, 100093 China; 2 Bureau of Landscape and Forestry, Mi Yun District, Beijing, 101500 China

## Abstract

A sublethal concentration of imidacloprid can cause chronic toxicity in bees and can impact the behavior of honey bees. The nectar- and water-collecting, and climbing abilities of bees are crucial to the survival of the bees and the execution of responsibilities in bee colonies. Besides behavioral impact, data on the molecular mechanisms underlying the toxicity of imidacloprid, especially by the way of RNA-seq at the transcriptomic level, are limited. We treated *Apis mellifera* L. with sublethal concentrations of imidacloprid (0.1, 1 and 10 ppb) and determined the effect on behaviors and the transcriptomic changes. The sublethal concentrations of imidacloprid had a limited impact on the survival and syrup consumption of bees, but caused a significant increase in water consumption. Moreover, the climbing ability was significantly impaired by 10 ppb imidacloprid at 8 d. In the RNA-seq analysis, gene ontology (GO) term enrichment indicated a significant down-regulation of muscle-related genes, which might contribute to the impairment in climbing ability of bees. The enriched GO terms were attributed to the up-regulated ribosomal protein genes. Considering the ribosomal and extra-ribosomal functions of the ribosomal proteins, we hypothesized that imidacloprid also causes cell dysfunction. Our findings further enhance the understanding of imidacloprid sublethal toxicity.

## Introduction

Imidacloprid is widely used against sucking pest insects because of its high selectivity and low toxicity to mammals^1^. When sprayed on or used as seed-coating agent on farmland, imidacloprid may kill some non-target organisms, such as pollinating insects and aquatic organisms^2–5^. Many organisms may be directly exposed to the imidacloprid spray, and systemic imidacloprid can easily diffuse to the plant tissues including pollens and nectar^6,7^. Bees are generally exposed to sublethal concentrations of imidacloprid in the environment, which impacts the behavior of honey bees (*Apis mellifera*). A sublethal concentration is defined as one that induces no statistically significant mortality in the experimental population. Sublethal effects are physiological or behavioral effects on individuals who survive an exposure to a pesticide whose dose/concentration can be sublethal or lethal^8^, whereas a non-observable-effect concentration (NOEC) is the highest concentration of a toxicant to which organisms are exposed during a test that causes no observable adverse effect on the test organisms^9^. In addition to their impact on learning and flying abilities, sublethal concentrations of imidacloprid also affect the feeding behaviors of bees^10–12^. Researchers have found that bees avoid feeding on a sugar solution spiked with imidacloprid, and this avoidance appears to be due to a repellent or anti-feedant effect^10–14^. Water collection and consumption, other distinctive behaviors of bees, are important tasks performed by the worker bees to satisfy their own needs, maintain necessary temperature and humidity, and mix with pollen to make brood food^15^. These two behaviors can be used as endpoints to evaluate the sublethal toxicity to bees and the ability to perform the tasks in a bee colony. Motor activity is also an important behavior of bees. In previous studies, the climbing abilities of bees and fruit flies were simply determined based on the detection of the ability to climb a certain distance within a certain time^16,17^. The effect of imidacloprid on the climbing ability of bees has not been studied yet. The climbing ability of bees is closely related to the performance of feeding, pollen and nectar collection, and nursing duties^15^.

Imidacloprid causes neurotoxicity in honey bees, and its primary target is the nicotinic acetylcholine (ACh) receptors that are present in the post-synaptic membranes of insect neurons^8,18–20^. Limited studies have been conducted investigating the molecular mechanisms underlying the side-effects of imidacloprid on honey bees at sublethal concentrations, especially by the way of RNA-seq at the transcriptomic level. Nauen *et al*. reported that imidacloprid binding site in honey bee head membrane preparations was consistent with that anticipated for a nAChR^21^. This interaction usually resulted in convulsions or paralysis of bees. However, paralysis from a loss of muscle function after imidacloprid treatment was lacking in bees. Bees are important bioindicators. We determined the survival, syrup- and water- consumption, and climbing behaviors of bees exposed to sublethal concentrations of imidacloprid. The purpose of the current study was to advance our understanding of exposure to sublethal concentrations of imidacloprid using bees as a model organism.

## Results

### Survival of bees and water and food consumption

Increasing concentration of imidacloprid and treatment time was applied to measure the effect of imidacloprid on experimental honey bee survival. Bees were exposed to 0, 0.1, 1, and 10 ppb imidacloprid and percent survival was determined at 1 d, 2 d, 4 d and 8 d. There was no significant decreased survival by 0.1, 1, or 10 ppb imidacloprid at all test time point (*P* > 0.05), which were from 93.67 ± 0.51% to 97.50 ± 1.53% including the control groups. The lowest survival percent (93.67 ± 0.51%) was in the group treated with 10 ppb imidacloprid at 8 d.

**Figure 1:**
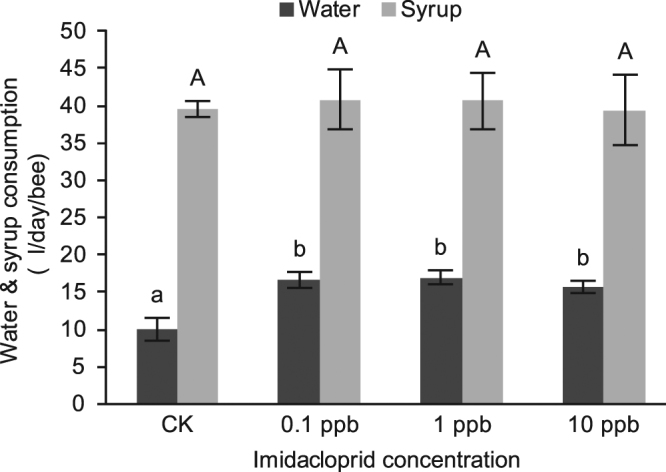
Daily water and syrup consumption of each bee after imidacloprid treatment. Values represent mean ± SEM. Significant differences from the control are indicated by different letters (*P* < 0.05).

Water and sucrose solution consumption were measured at each 24 h. Imidacloprid induced significant increased water consumption by honey bees (*P* < 0.05) when compared with the control groups, while there were no significant differences among 0.1, 1 and 10 ppb imidacloprid treatment (Fig. 1). These three concentrations of imidacloprid treatment had no effect on syrup consumption when compared with the control group (*P* > 0.05).

### Transcriptomic responses to sublethal concentration of imidacloprid

Three biological replicates were performed using RNA-seq sequencing for honey bee samples from treatment and control groups. The number of clean reads obtained from each of the six libraries after filtering and mapped the honey bee genome was about 30 million. A total of 13581 honey bee genes have been detected in each library. We then used the *P* value <0.05 and an absolute value for fold changes of >1.5 as criteria and 509 differentially expressed genes (160 up-regulated and 349 down-regulated genes) between control and imidacloprid treated groups (see supplemental Table S1).

### Validation of RNA-seq data by qRT-PCR

**Figure 2:**
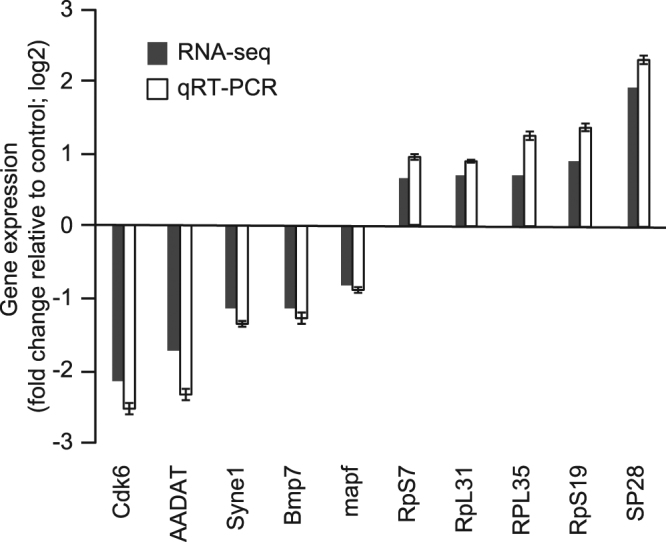
Verification of differentially expressed genes by qRT-PCR. Both ten different expressed genes with higher and lower expression in imidacloprid treated bees were randomly selected for qRT-PCR validation. The relative expression level of each gene was expressed as the fold change (log_2_) between lines in the RNA-seq data (black bar) and qRT-PCR data (white bar). Values represent mean ± SEM.

qRT-PCR assays using samples from a separate exposure experiment, which was conducted using the same exposure protocol as RNA-seq assays, were performed to confirm the results observed during RNA-seq analysis. The ten randomly selected genes (5 up-regulated and 5 down-regulated genes) showed the same expression profiles in the qRT-PCR assays in the RNA-seq data (Fig. 2).

### Muscle related genes implicated in imidacloprid sublethal exposure

**Figure 3:**
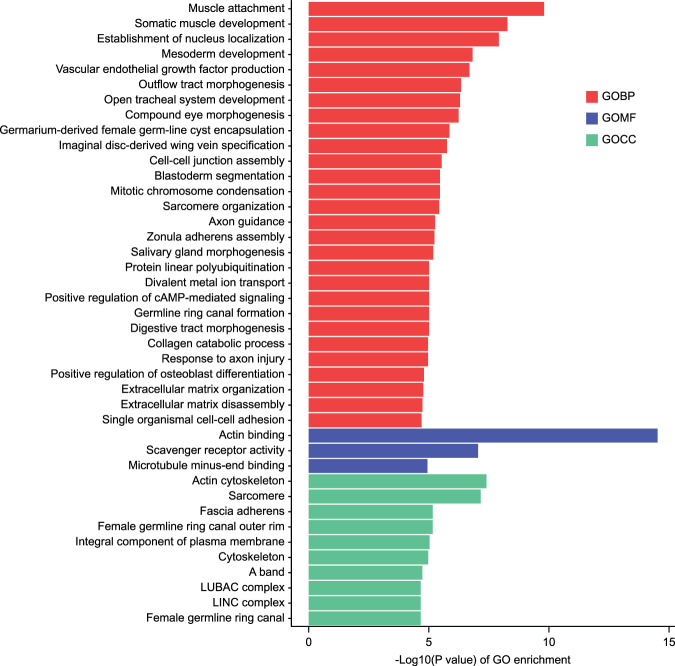
Significantly enriched Gene ontology (GO) terms (*P* < 0.05) in the down-regulated genes in imidacloprid treated bees. GO terms belong to biological processes (GOBP), molecular functions (GOMF), and cellular components (GOCC) are shown in red, green, and blue, respectively.

Gene ontology (GO) term enrichment analysis (*P* < 0.05) was performed to further understand the function of these differently expression genes. For down-regulated genes, the most significant enriched GO terms were “muscle attachment” (p = 5.52 E -5) in the biological process (GOBP) group, “actin binding” (p = 4.90 E -7) in the molecular function (GOMF) group and “actin cytoskeleton” (p = 6.09 E -4) in the cellular component (GOCC) (Fig. 3). Twenty-two genes that were related to the function of muscle were found to be repressed in the imidacloprid (10 ppb) treated bees. Amongst these repressed genes, 6 are muscle attachment related genes; 9 are actin binding related genes; and 3 are Ttn-like genes (see supplemental Table S2).

### Climbing behavior changes

**Figure 4:**
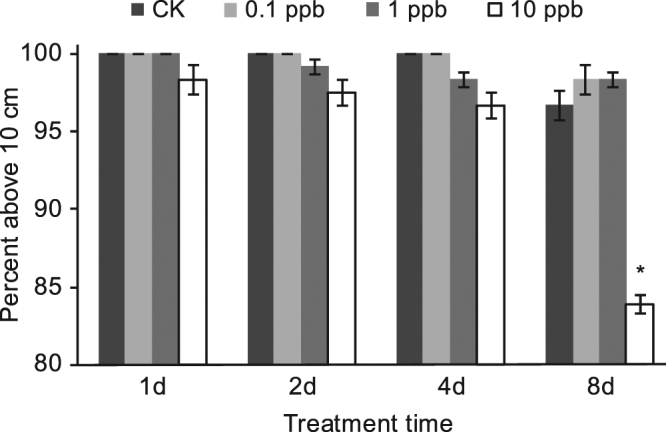
Geotaxis climbing ability of bees after imidacloprid treatment. Values represent mean ± SEM. Significant differences from the control are indicated by (*P* < 0.05).

Bees above the 10 cm line at the end of the 15 -second time period were counted (Fig. 4). The percentage of bees above the line that had been treated with 0.1 or 1 ppb imidacloprid was nearly 100% at all time points, and bees treated with 10 ppb were also nearly 100% at 1 d, 2 d, and 4 d. No significant decrease in climbing was observed among these imidacloprid treatments and control groups (*P* > 0.05). However, climbing was significantly impaired by 10 ppb imidacloprid at 8 d (83.87 ± 0.64%, p = 0.02).

### Ribosomal proteins were up-regulated by imidacloprid

**Figure 5:**
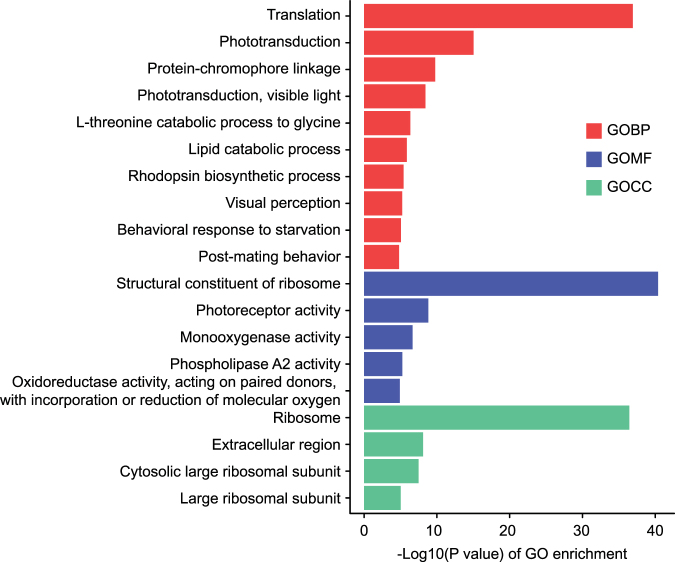
Significantly enriched GO terms (*P* < 0.05) in the up-regulated genes in imidacloprid treated bees. GO terms belong to biological processes (GOBP), molecular functions (GOMF), and cellular components (GOCC) are shown in red, green, and blue, respectively.

For up-regulated genes, the most significantly enriched GO terms were “translation” (p = 8.64 E -17) in the biological process (GOBP) group, “structural constituent of ribosome” (p = 2.46 E -18) in the molecular function (GOMF) group and “ribosome” (p = 1.65 E -16) in the cellular component (GOCC) (Fig. 5), respectively. Twenty-eight ribosomal protein genes were overexpressed in imidacloprid treatment groups, which included 18 60S ribosomal proteins and 10 40S ribosomal proteins (see supplemental Table S3).

## Discussion

Honey bees are important pollinator insects^22–24^. To investigate the effects of sublethal concentrations of imidacloprid (0.1 to 10 ppb) on honey bees, we studied both the behaviors and molecular mechanism after oral exposure to imidacloprid-treated syrup. We confirmed that concentrations of imidacloprid below 10 ppb had no significant effect on survival and syrup consumption, but water consumption was significantly increased (up to 63.38% more on average based on the results shown in Fig. 1). GO term enrichment analysis of RNA-seq data indicated a down-regulation of muscle-related genes, as well as a significant reduction in the climbing ability of bees (10 ppb, 8 d). Moreover, the ribosomal protein genes were significantly up-regulated.

Sublethal concentrations of imidacloprid had a minor impact on the survival of bees (0.1, 1, and 10 ppb; 1, 2, 4, and 8 d), and our results confirmed that imidacloprid concentrations below 20 ppb are not lethal to honey bees^25,26^. In addition, there were no significant differences in the daily syrup consumption of each bee (Fig. 1). It seemed that no avoidance of feeding occurred at such concentration (≤10 ppb, 8 d). Thus, the impact of imidacloprid on bees in this study might be not related to anti-feedant effects or weakness from reduction of syrup consumption. It has been reported that bees avoided feeding when the peak concentration of imidacloprid was approximately 30 ppb^10^. Our study provided supplemental data under a low concentration (≤10 ppb, 8 d). Moreover, the daily water consumption of the bees increased after imidacloprid treatment. This finding is in agreement with a previous report on a different insecticide, fipronil; the sublethal concentrations of fipronil caused a significant increase in the daily amount of water consumed^16^. The only difference was that fipronil had a slightly greater toxicity on bees and caused more deaths (approximately 3 ppb, 8 d, 20% of mortality) than imidacloprid (10 ppb, 8 d, 8% mortality). This observation may be explained as an effort to relieve the imidacloprid-induced thirst or to dilute the imidacloprid ingested by the bees. Further studies are needed to confirm this finding.

However, there were no significant differences in the amount of water consumed by bees exposed to thiamethoxam and acetamiprid under the same conditions, compared with the control group^16^. Moreover, none of the test concentrations (0.1, 1, and 10 ppb) used in this study induced statistically significant mortality in the experimental bees, so they were considered sublethal concentrations. In addition, 0.1 and 1 ppb of imidacloprid (except for the effect of 10 ppb on climbing ability) did not cause adverse effects on the bees in this study, which are specifically non-observable effect concentrations (NOECs)^25,26^. Therefore, bee colonies exposed to NOECs of insecticides may survive and appear healthy, while the potential adverse impact on individual bees is neglected. This might lead to considerable loss of the bee colonies at a later time. It is known that imidacloprid and other neonicotinoids have delayed and enhanced time–dependent chronic toxicity. To estimate the effect of chronic pesticide exposure on honey bees, chronic tests for pesticide toxicity to pollinators should be extended to 30 days or more^27^. Pesticides that exhibit enhanced toxicity require much more care for the toxin to be fully-eliminated from the pollinators’ environment. Thus, there is an urgent need to revise methods of assessing the risks of systemic pesticides to bees^8,27,28^.

Imidacloprid is a key neonicotinoid insecticide. It acts as an agonist of acetylcholine to occupy the binding site of nicotinic ACh receptors in the central nervous system causing excitation and eventually paralysis leading to death^18–20^. In the current study, transcriptome sequencing validated the down- regulation of muscle-related genes to which the enriched GO terms were attributed. The smallest building block of a muscle is the sarcomere. Sarcomeres are mainly made up of actin and filaments. Muscle shortening or contraction depends on these filaments sliding against each other, and requires that the actin filaments are anchored in Z- discs. The major Z- disc protein is α- actinin, which is also responsible for anchoring another protein called Ttn (titin). Ttn ensures that the actin and myosin filaments of a sarcomere are positioned correctly and re-adjusts the sarcomere to its original length after muscle activity^29–32^. The decreased expression of the genes which regulate muscle function in this study suggest that muscle contraction or movement function might be reduced by the down-regulation of these genes (muscle attachment, actin binding- related genes, and Ttn- like genes).

We performed a climbing test on the bees. The results indicated a significant reduction in their climbing ability after 8 days exposure to 10 ppb, but not before. In addition to the neural effect, imidacloprid also inhibited muscular function. Imidacloprid itself did not cause muscle cell contractions but at concentrations ≥3.3 E -6 M prevented or attenuated those induced by acetylcholine (5 E -7 M and 5 E -5 M) or nicotine (5 E -6 M to 5 E -3 M) in embryonic frog muscle cells^33^. It suggests that imidacloprid is an antagonist at the nAChR in muscle cells in contrast to its established role as an agonist in its toxic action on insect neural receptors. We inferred that the effects of imidacloprid involved the following: interfering with neuro-transmission in the central nervous system, inhibiting neuronal nAChR, and down-regulating the expression of muscle-related genes. These effects might jointly cause the impairment of the climbing ability of bees. The down-regulation of muscle-related genes was reported for the first time in this study, and more direct and convincing evidence is needed for validation as honey bees exhibit complex behavioral patterns^17^.

The findings of this study also indicated that sublethal concentrations of imidacloprid induced the up-regulation of ribosomal protein genes in bees. Ribosomal proteins are among the most highly conserved proteins across all life forms. In addition to performing protein synthesis in the cell in coordination with rRNA, ribosomal proteins have extra-ribosomal functions in cellular processes such as DNA repair (e.g., P0), replication (e.g., S1, S14, L14), RNA processing (S14, L4, L22), transcription (e.g., S1, S14, L4, L14, S7), and development (e.g., S6, S19)^34–36^. The up-regulation of several ribosomal proteins has been shown in human cancer (e.g., S6, P0, L5, L7a, L13, L35a, S7, S8, S29)^36^. In addition, overexpression of ribosomal protein L22 in *Drosophila* cells resulted in global suppression of transcriptional genes^37^. Most of the genes mentioned above were up-regulated in this study (see supplemental information Table S1). The up-regulation of the ribosomal protein genes may contribute to cell dysfunction; however, concrete evidence from further experiments is needed to support this view.

To summarize, sublethal concentrations (0.1 to 10 ppb) of imidacloprid had a limited impact on the survival and syrup consumption of bees, but caused a significant increase in water consumption. RNA-seq analysis indicated the down-regulation of muscle-related genes in bees exposed to imidacloprid. Based on the results of our climbing ability assay and a literature review, we inferred that the down-regulation of muscle-related genes caused by imidacloprid might contribute to the impairment of the climbing ability. Moreover, the ribosomal protein genes were up-regulated. Considering the ribosomal and extra-ribosomal functions of the ribosomal proteins, imidacloprid might cause cell dysfunction as well. Bees are important model organisms. The molecular changes caused by sublethal concentrations of imidacloprid may predict the potential impact on motor ability and normal cell functioning of bees in addition to the neural effects.

## Materials and Methods

### Insects and chemical preparation

Experiments were conducted with colonies of *A. mellifera ligustica* maintained at the Institute of Apicultural Research apiary, Chinese Academy of Agricultural Science during summer (July-September 2015).

A sanitary control was carried out on the colonies, and the treatment ended at least 4 weeks before the start of the experiments. Frames of 3–5 different colonies with capped worker brood were placed in an incubator. Newly emerged workers were removed from each frame by gently shaking the frame over a paper box (30 × 30 × 10 cm) within 12 h of their emergence. Bees were cold-anesthetized at 4 °C for 5 min before being placed in rearing cages (15 × 15 × 10 cm; 40 workers per cage) with mesh on two sides. Cages were kept in an incubator at 33 ± 1 °C and 45 ± 5% relative humidity in darkness. Plastic feeders with eight separate feeding tubes (300 ul for each tube) were inserted vertically into the cages and changed daily. Honey bees were provided with pollen, water, and sucrose solution (50% wt/wt) ad libitum for the first week and were then subjected to an 8 d exposure period as described below.

### Imidacloprid exposure protocols

Honey bees were exposed to 0.1, 1, or 10 ppb imidacloprid and collected at 1 d, 2 d, 4 d and 8 d. Imidacloprid (99.99% purity) was dissolved in acetone and diluted in a sucrose solution (50% wt/wt). The final concentration of acetone in the sucrose solutions was equal to 0.03% (vol./vol.). The sucrose solution used for feeding the bees contained imidacloprid for oral treatment and acetone for control groups. The sublethal concentrations of the compound used here were based on previous studies^38,39^. Two feeders were provided: (1) one feeder of two tubes with deionized water, and (2) one feeder of eight tubes with 1 M sucrose containing a specific concentration of imidacloprid (0.1, 1, and 10 ppb) or acetone (for control) ad libitum, which were changed daily^40,41^. In all, 30 cages of honey bees were prepared, and 12 cages (three replicates for each concentration including control) were randomly selected to be used for the climbing assay. An additional 12 cages were used to measure the survival rate, sucrose and water consumption, whereas the remaining cages were used for RNA-seq detection.

### Lethality and syrup and water consumption assay

Live honey bees were counted at 1 d, 2 d, 4 d and 8 d. Lethality was expressed as the percentage of surviving honey bees compared to the number of live bees at the beginning of the experiment. Feeding tubes with sucrose (0, 0.1, 1 and 10 ppb imidacloprid) were weighed, placed in the experimental boxes with the bees at each time point, and then removed and weighed a second time as described by Kessler *et al*. for measuring the amount of syrup consumption^42,43^. Control boxes identical to the experimental boxes (without bees) for each imidacloprid treatment were placed in the incubator simultaneously with the food solutions experiments. The amount of solution consumed was defined as the difference in the weight of each feeder, and the amount was divided by the number of surviving bees at each 24 h interval; the average value for the evaporation control for each treatment was subtracted from this final value for each feeder. Water was in a separate feeder and was measured using the same method as the syrup consumption assay.

### RNA isolation and sequencing

Honey bees that survived exposure to the greatest concentration of imidacloprid (10 ppb) for each time point (1 d, 2 d, 4 d and 8 d) were collected (10 bees from one of three cages at each time point) for transcriptomic analysis. There were three biological replicates each for the control and imidacloprid-treated groups. Total RNA was extracted from pools of honey bees (taken at each of the individual time points) using TRIzol reagent following the manufacturer’s instruction. Possible RNA degradation and contamination were checked on 1% agarose gels. RNA purity was determined using the NanoDrop 2000 and the RNA integrity and concentration were evaluated by Agilent 2100 RNA Nano 6000 Assay Kit (Agilent Technologies, Inc., Santa Clara, CA, USA). Only RNA samples which passed the quality tests were used for RNA-Seq analyses. mRNA was enriched using magnetic beads with Oligo (dT), and then fragmented in 1× NEB Next Magnesium RNA Fragmentation Buffer. The shorted mRNA fragments were used as templates, and first-strand cDNA was synthesized using random hexamers. Next the second-strand cDNA was synthesized using dNTPs, DNA polymerase I and RNAaseH. The double-strand cDNA was purified by QIAQuick PCR kit. cDNA was then used for end reparation, “A” base addition and ligated with sequencing adapters. The AMPure XP beads system (Beckman Coulter, Beverly, USA) was used to select cDNA fragments. After quantification with Agilent Bioanalyzer 2100 system, cDNAs were used for PCR amplication and sequenced with an Illumina Nextseq. 500 platform in a 150 bp paired-end pattern.

### Bioinformatics analysis of RNA-seq data

Raw reads were pre-processed to remove low quality regions and adapter sequences. Sequence tag preprocessing was performed according to a previously described protocol with some modification^44^. Reads with adaptors of low quality (>50%) or a high -proportion of unknown bases (>5%) were removed. Clean data were mapped to the *A. mellifera genome* (version Amel_4.5 downloaded from NCBI) using TopHat software with a maximum allowance of 2 nucleotide mismatches.

### Gene Ontology (GO) term enrichment analysis

Functional enrichment analyses were performed to identify which differentially expressed genes were significantly enriched in GO terms. Parent-Child-Intersection method was used for enrichment analysis and Benjamini-Hochberg was used for multiple tests correction, curated association was used for enrichment analysis. Go terms with corrected *P* value less than 0.05 were considered significantly enriched by differentially expressed genes.

### Quantitative real-time polymerase chain reaction (qRT-PCR)

qRT-PCR analyses were performed with independently generated samples from a separate exposure experiment conducted using the same exposure protocol as describe above. Extraction of total RNA from pooled honey bee samples, first-strand cDNA syntheses and qRT-PCR were performed using commercial kits as described previously^45,46^. Briefly, the extraction of total RNA was performed using TRIzol regent following manufacturer’s instructions. RNA concentration and quality were assessed using the NanoDrop 2000. The reaction mixture and thermal cycling profiles of cDNA synthesis were performed as previously described^45,46^. qRT-PCR was performed using a SYBR Green-based KAPA FAST qPCR Kit (Kapa Biosystems, Boston, MA, USA), following manufacturer’s instructions. Primers were designed using Primer 5 software (http://bioinfo.ut.ee/primer3-0.4.0/primer3/) (see supplement information Table S4). β-actin gene was used as housekeeping gene to normalize results to minimize variation between and among analyses. Thermal cycling was set at 95 °C for 3 min, followed by 40 cycles of 94 °C for 3 s and 60 °C for 30 s. qRT-PCR data were presented as fold change (log_2_) relative to control. Each qRT-PCR experiment was repeated three times. The relative expression of genes were normalized by comparison with the expression of β-actin, and analyzed using the 2^−ΔΔC^T Method^47^.

### Climbing assays

Muscle and actin related genes are involved in motor activities of the bees. To further survey the ability of climbing, geotactic climbing assay was used to examine the effects of imidacloprid on honey bees. The climbing assay was modified from the protocol described by Podratz *et al*. and Lemmon^17,48^. From each cage, 10 bees were placed into an empty plastic box (10 × 5 × 15 cm) (except for the glass front allowing observation) with a mark placed 10 cm from the bottom and a fluorescent lamp in the top. The tests were performed in dark and with the lamp turned on. This stimulated locomotion in the bees by positive phototaxis. The bees were gently knocked to the bottom of the box by tapping on the top. The box was then stood vertically, and recording of the time when all bees were at the bottom was initiated. The number of bees that climbed above the 10 cm line was recorded after 15 seconds. This was repeated 5 times per box with 3 boxes per condition. Climbing ability was expressed as a percentage of the number of bees above the 10 cm line as compared to the total number of bees.

### Statistical analyses

One-way analysis of variance (ANOVA) was used for comparing responses between treatment groups for survival, water and syrup consumption, climbing ability, and gene expression (qRT-PCT). Statistical significance was analyzed using the Tukey-Kramer multiple comparision post-ANOVA test. IBM SPSS Statistics 19 (IBM Corp., Armonk, NY) was used to conduct all statistical analyses. *P* < 0.05 was considered statistically significant.

## Electronic supplementary material

Supplementary information
