# Supplementary material for: Sublethal effects of imidacloprid on targeting muscle and ribosomal protein related genes in the honey bee Apis mellifera L
Source: Sci Rep. 2017 Nov 21;7:15943. doi: 10.1038/s41598-017-16245-0 (PMC5698328; doi:10.1038/s41598-017-16245-0)
Supplement: Supplementary file 1 — Supplementary information [file 41598_2017_16245_MOESM1_ESM.pdf]

Supplementary information for following article

Sublethal effects of imidacloprid on targeting muscle and ribosomal protein related genes in the honey bee *Apis mellifera* L.

Yan-Yan Wu[1], Qi-Hua Luo[2], Chun-Shen Hou[1], Qiang Wang[1], Ping-Li Dai[1], Jing Gao[1], Yong-Jun Liu[1],Qing-Yun Diao[1,]

[1]Key Laboratory of Pollinating Insect Biology, Ministry of Agriculture, Institute of Apicultural Research, Chinese Academy of Agricultural Sciences, Beijing 100093, China
[2] Bureau of Landscape and Forestry, Mi Yun District, Beijing 101500, China
[*]Corresponding author:
Qing-Yun Diao (Q.Y. Diao)
Key Laboratory of Pollinating Insect Biology, Ministry of Agriculture,
Institute of Apicultural Research, Chinese Academy of Agricultural Sciences,
Beijing 100093,
China
Tel: +8610 62597285
Fax: +8610 62597285
Email: dqyun1@126.com

Table S1. 509 differentially expressed genes between control and imidacloprid treated groups

<table>
<tr><th>Gene</th><th>SIM_normalize</th><th>SCK_normalize</th><th>FoldChange</th><th>Log_2Fold Change</th><th>pval</th><th>NR:Seq-id</th><th>NR:Description</th></tr>
<tr><td>gene3689</td><td>1003.918525</td><td>264.3827841</td><td>3.797215951</td><td>1.924942048</td><td>0.000136</td><td>gi|571524910|ref|XP_006567203.1|</td><td>PREDICTED: venom serine protease 34 isoform X1 [Apis mellifera]</td></tr>
<tr><td>gene12324</td><td>125.3372686</td><td>35.67690611</td><td>3.513120455</td><td>1.812753043</td><td>0.000349</td><td>gi|328793159|ref|XP_625207.2|</td><td>PREDICTED: phospholipase B1, membrane-associated-like isoform X3 [Apis mellifera]</td></tr>
<tr><td>gene6640</td><td>4062.789507</td><td>1436.279536</td><td>2.828689963</td><td>1.500134059</td><td>2.24E-10</td><td>gi|118150502|ref|NP_001071288.1|</td><td>chemosensory protein 1 precursor [Apis mellifera]</td></tr>
<tr><td>gene11505</td><td>38.4053804</td><td>13.61559682</td><td>2.820690192</td><td>1.496048218</td><td>0.018742</td><td>gi|118150512|ref|NP_001071293.1|</td><td>long wavelength sensitive opsin 2 [Apis mellifera]</td></tr>
<tr><td>gene6088</td><td>213.4106065</td><td>81.05746998</td><td>2.632830836</td><td>1.396614829</td><td>0.007057</td><td>gi|110758297|ref|XP_001120293.1|</td><td>PREDICTED: phospholipase A2-like [Apis mellifera]</td></tr>
<tr><td>gene4856</td><td>299.842302</td><td>117.1373937</td><td>2.559748791</td><td>1.356002234</td><td>0.001202</td><td>gi|571532643|ref|XP_006558886.1|</td><td>PREDICTED: uncharacterized protein LOC726672 [Apis mellifera]</td></tr>
<tr><td>gene4913</td><td>3164.636073</td><td>1246.956822</td><td>2.537887452</td><td>1.343628091</td><td>0.019333</td><td>gi|58585164|ref|NP_001011608.1|</td><td>alpha-glucosidase precursor [Apis mellifera]</td></tr>
<tr><td>gene622</td><td>7752.783798</td><td>3058.102863</td><td>2.535161224</td><td>1.342077499</td><td>0.000213</td><td>gi|110749126|ref|XP_394209.2|</td><td>PREDICTED: glucose dehydrogenase [FAD, quinone] isoform 3 [Apis mellifera]</td></tr>
<tr><td>gene3</td><td>2607.675</td><td>1055.878</td><td>2.4696</td><td>1.3043209</td><td>0.000</td><td>gi|328789967|ref</td><td>PREDICTED: laccase-5-like [Apis mellifera]</td></tr>
</table>

117	9	357	74544	35	835	|XP_625189.2|	
gene11504	5489.111853	2268.534987	2.419672557	1.274811828	0.001881	gi|283135098|ref|NP_001011639.2|	rhodopsin, long-wavelength [Apis mellifera]
gene9572	49.36412267	20.67232648	2.387932616	1.255762127	0.003714	gi|209969890|ref|NP_001129681.1|	silk fibroin 4 precursor [Apis mellifera]
gene4229	38.09507864	15.9800568	2.383913843	1.253332097	0.031971	gi|645015869|ref|XP_001601386.2|	PREDICTED: 40S ribosomal protein S29 [Nasonia vitripennis]
gene11561	127.6784653	54.42497435	2.345953614	1.230174488	4.22E-05	gi|571574902|ref|XP_006563622.1|	PREDICTED: uncharacterized protein LOC100576330 [Apis mellifera]
gene8291	4004.018412	1712.763153	2.337753709	1.225122944	0.00836	gi|66517414|ref|XP_623184.1|	PREDICTED: arrestin homolog isoform 2 [Apis mellifera]
gene7311	860.7985781	372.6899935	2.309690609	1.207699611	0.005136	gi|820836729|ref|XP_012346672.1|	PREDICTED: protein takeout-like [Apis florea]
gene918	4243.347557	1870.714798	2.26830277	1.181613222	0.031592	gi|66500020|ref|XP_397545.2|	PREDICTED: uncharacterized protein LOC409096 [Apis mellifera]
gene13571	35.25667399	15.55119925	2.267135378	1.180870542	0.04774	gi|571579756|ref|XP_006572785.1|	PREDICTED: alanine--glyoxylate aminotransferase 2-like [Apis mellifera]
gene7564	1220.091073	546.6287754	2.232028624	1.158355529	0.000162	gi|58585118|ref|NP_001011589.1|	odorant binding protein 4 precursor [Apis mellifera]

gene8767	127.0497722	58.19953832	2.183003093	1.126314175	0.006367	gi|571558341|ref|XP_006564092.1|	PREDICTED: pancreatic triacylglycerol lipase-like [Apis mellifera]
gene11344	94.33901449	43.29287892	2.179088498	1.123724789	0.000918	gi|572298283|ref|XP_006615299.1|	PREDICTED: uncharacterized protein LOC102672709 [Apis dorsata]
gene4646	310.3408783	145.4367074	2.133855228	1.093462299	0.00762	gi|328790707|ref|XP_001120503.2|	PREDICTED: transient receptor potential protein [Apis mellifera]
gene6556	30.09473295	14.22860368	2.115086879	1.080716924	0.033822	gi|571543586|ref|XP_006561486.1|	PREDICTED: ras-like protein family member 11B-like [Apis mellifera]
gene9140	62.24895922	29.76868756	2.091088467	1.064254099	0.006037	.	.
gene4514	106.5166367	50.98274518	2.089268366	1.062997818	0.003774	gi|110756792|ref|XP_001120695.1|	PREDICTED: uncharacterized protein LOC726118 [Apis mellifera]
gene6036	355.1340028	170.7369657	2.080006526	1.056588055	0.002322	gi|571539762|ref|XP_006565521.1|	PREDICTED: phospholipase B1, membrane-associated-like isoform X1 [Apis mellifera]
gene1683	254.9297916	122.8177222	2.075675946	1.053581228	0.024035	gi|218156311|ref|NP_001136165.1|	uncharacterized protein LOC100217360 [Apis mellifera]
gene12058	918.2284519	446.4724505	2.056629588	1.040281979	0.016952	gi|58585212|ref|NP_001011635.1|	yellow-f precursor [Apis mellifera]

gene10921	39.0892373	19.01873452	2.055301695	1.039350181	0.02081	gi|820852399|ref|XP_003694757.2|	PREDICTED: uncharacterized protein LOC100869988 [Apis florea]
gene11723	73.50686947	35.88240541	2.048549105	1.034602475	0.012804	gi|571575711|ref|XP_006557840.1|	PREDICTED: elongation of very long chain fatty acids protein AAEL008004-like isoform X2 [Apis mellifera]
gene6728	1455.333317	713.0494787	2.040999062	1.029275519	0.000424	gi|328784765|ref|XP_624408.2|	PREDICTED: trans-1,2-dihydrobenzene-1,2-diol dehydrogenase-like [Apis mellifera]
gene10514	1140.489415	567.542659	2.009521922	1.006852316	0.016271	gi|201023353|ref|NP_001128419.1|	esterase A2 [Apis mellifera]
gene10882	199.2520175	99.87751751	1.994963656	0.996362464	0.033733	gi|58585160|ref|NP_001011606.1|	opsin, blue-sensitive [Apis mellifera]
gene6400	250.2774917	125.5646715	1.993215837	0.995097942	5.42E-05	gi|110773269|ref|XP_001120568.1|	PREDICTED: cytochrome c oxidase subunit 7C, mitochondrial-like [Apis mellifera]
gene11045	776.9336862	392.1976598	1.980974814	0.986210538	3.16E-06	gi|94158709|ref|NP_001035295.1|	odorant binding protein 16 precursor [Apis mellifera]
gene10777	188.2840331	95.07633037	1.980346026	0.985752535	0.000113	gi|328785069|ref|XP_001121591.2|	PREDICTED: uncharacterized protein LOC725784 isoformX1 [Apis mellifera]
gene10174	73.56363886	37.36558115	1.968754041	0.977282884	0.005558	gi|571567810|ref|XP_391975.4|	PREDICTED: peptidyl-prolyl cis-trans isomerase, rhodopsin-specific isozyme-like isoform X2 [Apis mellifera]
gene1	184.1792	93.89785	1.9614	0.9719467	0.000	gi|110763495|ref	PREDICTED: MORN repeat-containing protein 4 [Apis mellifera]

1400	861	348	85585	33	129	|XP_393307.3|	
gene11346	105.1018008	53.82203235	1.952765368	0.965518615	0.029484	gi|328783471|ref|XP_001120999.2|	PREDICTED: chymotrypsin inhibitor-like isoform 1 [Apis mellifera]
gene13483	118.3173119	60.69339245	1.94942657	0.963049813	0.004026	gi|66548280|ref|XP_624540.1|	PREDICTED: dehydrogenase/reductase SDR family member 11-like isoform X2 [Apis mellifera]
gene10317	1179.720156	606.6855157	1.944533247	0.959423902	0.000958	gi|66523006|ref|XP_396534.2|	PREDICTED: probable cytochrome P450 6a14 isoformX1 [Apis mellifera]
gene6339	118.4171544	61.18218154	1.935484342	0.952694636	0.002023	gi|572303416|ref|XP_006617694.1|	PREDICTED: uncharacterized protein LOC102676732 [Apis dorsata]
gene572	833.5277943	432.1252193	1.928903376	0.947780877	0.000354	gi|571502919|ref|XP_006565388.1|	PREDICTED: uncharacterized protein LOC413056 [Apis mellifera]
gene1682	104.4473239	54.4900232	1.916815551	0.938711518	0.044312	gi|820859691|ref|XP_003697124.2|	PREDICTED: tetraspanin-7-like [Apis florea]
gene11831	242.167113	126.6310374	1.912383551	0.935371902	0.015554	gi|328778410|ref|XP_001120045.2|	PREDICTED: homeotic protein deformed [Apis mellifera]
gene11347	55.20096445	29.06555456	1.899188413	0.925383038	0.036417	gi|571574368|ref|XP_006563415.1|	PREDICTED: mucin-6-like isoform X1 [Apis mellifera]
gene12112	428.9224056	228.5108154	1.877033281	0.90845423	4.04E-05	gi|66526281|ref|XP_396852.2|	PREDICTED: scavenger receptor class B member 1 [Apis mellifera]
gene8	410.0094	219.1903	1.8705	0.9034728	0.000	gi|66534390|ref|	PREDICTED: pancreatic triacylglycerol lipase-like isoform X2 [Apis mellifera]

768	572	505	63445	99	127	XP_623663.1|	
gene13055	825.6577386	441.7616678	1.869011729	0.902275623	2.71E-06	gi|759079256|ref|XP_011349442.1|	PREDICTED: 40S ribosomal protein S19-like isoform X1 [Cerapachys biroi]
gene5792	1438.974909	776.1995504	1.853872382	0.890541934	7.32E-07	gi|307173970|gb|EFN64700.1|	40S ribosomal protein S15 [Camponotus floridanus]
gene8719	119.5378144	64.93917443	1.840765847	0.880306122	0.003015	gi|571558039|ref|XP_006562102.1|	PREDICTED: tetratricopeptide repeat protein 39C-like [Apis mellifera]
gene11350	1125.545578	615.3632538	1.829075057	0.871114278	0.015569	gi|571574380|ref|XP_006563420.1|	PREDICTED: chymotrypsin inhibitor-like isoform X2 [Apis mellifera]
gene10988	857.1372661	469.5715307	1.825360377	0.86818132	2.12E-06	gi|571573277|ref|XP_006557994.1|	PREDICTED: CDGSH iron-sulfur domain-containing protein 2 homolog isoform X1 [Apis mellifera]
gene11340	171.75113	94.40159593	1.819366805	0.863436436	0.028863	gi|110763428|ref|XP_001120193.1|	PREDICTED: uncharacterized protein LOC725232 [Apis mellifera]
gene13333	51.82689664	28.52126477	1.81713178	0.861663049	0.027959	gi|48097526|ref|XP_391912.1|	PREDICTED: alpha-tocopherol transfer protein-like [Apis mellifera]
gene11095	132.621246	73.3819813	1.807272626	0.853814152	0.042945	gi|66548280|ref|XP_624540.1|	PREDICTED: dehydrogenase/reductase SDR family member 11-like isoform X2 [Apis mellifera]
gene1641	272.5564694	151.6818442	1.796895804	0.845506754	0.027448	gi|571510600|ref|XP_006571771.1|	PREDICTED: uncharacterized protein LOC102654257 [Apis mellifera]
gene1	1021.181	572.6309	1.7833	0.8345619	9.09E	gi|328779527|ref	PREDICTED: uncharacterized protein LOC552366 [Apis mellifera]

95	668	468	15543	99	-06	|XP_625285.2|	
gene1<br>2553	78.62719<br>841	44.18166<br>937	1.7796<br>33941	0.8315805<br>19	0.016<br>661	.	.
gene2<br>375	121.2298<br>602	68.12836<br>747	1.7794<br>32925	0.8314175<br>52	0.010<br>035	gi|571514717|ref<br>|XP_006568869.<br>1|	PREDICTED: uncharacterized protein LOC724555 isoform X1 [Apis mellifera]
gene8<br>822	536.1067<br>392	302.3450<br>366	1.7731<br>62031	0.8263243<br>76	0.002<br>126	gi|380029575|ref<br>|XP_003698444.<br>1|	PREDICTED: 60S ribosomal protein L32 [Apis florea]
gene8<br>005	92.50236<br>257	52.44422<br>398	1.7638<br>2365	0.8187063<br>25	0.009<br>813	gi|328784573|ref<br>|XP_001122994.<br>2|	PREDICTED: serendipity locus protein H-1-like [Apis mellifera]
gene3<br>674	49959.67<br>741	28489.18<br>165	1.7536<br>36802	0.8103499<br>8	0.002<br>401	gi|58585154|ref| NP_001011607.<br>1|	melittin precursor [Apis mellifera]
gene1<br>1047	2087.784<br>93	1192.114<br>72	1.7513<br>28874	0.8084500<br>26	0.035<br>956	gi|94158830|ref| NP_001035317.<br>1|	odorant binding protein 18 precursor [Apis mellifera]
gene7<br>65	502.4862<br>996	287.9221<br>767	1.7452<br>15688	0.8034053<br>47	7.49E<br>-05	gi|571503975|ref<br>|XP_006568688.<br>1|	PREDICTED: uncharacterized protein LOC408592 isoform X1 [Apis mellifera]
gene9<br>451	602.1048<br>77	346.5165<br>649	1.7375<br>93345	0.7970904<br>83	0.034<br>942	gi|571563280|ref<br>|XP_006558363.<br>1|	PREDICTED: facilitated trehalose transporter Tret1-like [Apis mellifera]
gene1<br>0415	569.8122<br>85	328.4087<br>086	1.7350<br>7057	0.7949943<br>42	0.012<br>279	gi|815768511|ref<br>|XP_012231503.	PREDICTED: uncharacterized protein LOC105677458 [Linepithema humile]

						1|	
gene7565	8968.287148	5173.314532	1.73356696	0.793743563	0.013815	gi|94158729|ref|NP_001035311.1|	odorant binding protein 3 precursor [Apis mellifera]
gene172	138.2338759	79.74826056	1.733377943	0.793586252	0.007465	gi|572270852|ref|XP_006613410.1|	PREDICTED: pre-mRNA-splicing factor CWC22 homolog isoform X1 [Apis dorsata]
gene4019	56.93668085	32.84762473	1.733357627	0.793569342	0.048287	gi|328780013|ref|XP_003249736.1|	PREDICTED: uncharacterized protein LOC100577683 [Apis mellifera]
gene4029	74.56387108	43.04345194	1.732293014	0.792682979	0.030887	gi|58585100|ref|NP_001011575.1|	nicotinic acetylcholine receptor alpha8 subunit [Apis mellifera]
gene5376	484.1611425	280.2891666	1.727363024	0.788571313	0.001927	gi|571535759|ref|XP_001119990.2|	PREDICTED: uncharacterized protein LOC724221 isoform 1 [Apis mellifera]
gene1044	58.53595719	33.94581154	1.724394102	0.786089533	0.046946	gi|733869626|ref|XP_010708157.1|	PREDICTED: histone H3-like [Meleagris gallopavo]
gene4760	61.93738044	35.9691512	1.721958355	0.784050252	0.045671	gi|380024952|ref|XP_003696250.1|	PREDICTED: transcription and mRNA export factor ENY2 [Apis florea]
gene3838	228.0182276	132.8743782	1.716043609	0.779086216	0.031991	gi|571525702|ref|XP_006567356.1|	PREDICTED: uncharacterized protein LOC102654949 [Apis mellifera]
gene1	73.32726	42.76249	1.7147	0.7780037	0.034	gi|571508217|ref	PREDICTED: uncharacterized protein LOC551357 isoform X1 [Apis mellifera]

294	278	197	5654	58	907	|XP_006571299.1|	
gene2309	146.4416058	85.79326133	1.706912682	0.771389258	0.042146	gi|571515131|ref|XP_006568941.1|	PREDICTED: soma ferritin [Apis mellifera]
gene10274	1276.8104	748.3118383	1.706254445	0.770832805	0.000994	gi|58585144|ref|NP_001011598.1|	alpha-amylase precursor [Apis mellifera]
gene1434	252.641377	148.2108179	1.70460821	0.769440186	0.013995	gi|110766219|ref|XP_001120479.1|	PREDICTED: ribonuclease kappa-like [Apis mellifera]
gene2279	72.27949484	42.42015139	1.703895259	0.768836654	0.023574	gi|328789556|ref|XP_003251294.1|	PREDICTED: uncharacterized protein LOC100576118 isoform X1 [Apis mellifera]
gene1484	80.96610154	47.57380305	1.701905174	0.767150655	0.016207	gi|201023329|ref|NP_001128406.1|	GABA neurotransmitter transporter-1A [Apis mellifera]
gene681	129.7908969	76.56575205	1.695156038	0.761418079	0.021704	gi|48138800|ref|XP_396933.1|	PREDICTED: cell death-related nuclease 6 isoform X1 [Apis mellifera]
gene11923	4183.135548	2472.690294	1.691734528	0.758503194	1.17E-05	gi|66526748|ref|XP_624696.1|	PREDICTED: 60S ribosomal protein L27a isoform X2 [Apis mellifera]
gene8055	89.32091513	52.96326673	1.686469144	0.754005923	0.017993	gi|571553698|ref|XP_006565579.1|	PREDICTED: uncharacterized protein LOC725578 isoform X1 [Apis mellifera]
gene661	119.4236047	70.88404328	1.684774164	0.752555218	0.01282	gi|328788801|ref|XP_001122217.	PREDICTED: facilitated trehalose transporter Tret1-like [Apis mellifera]

						2|	
gene10792	211.9464421	126.4379328	1.676288416	0.745270395	0.034822	gi|328785089|ref|XP_394000.3|	PREDICTED: carotenoid isomerooxygenase isoform 1 [Apis mellifera]
gene7304	2040.04696	1225.067773	1.665252327	0.735740798	0.001374	gi|328781001|ref|XP_393105.3|	PREDICTED: circadian clock-controlled protein-like isoform 1 [Apis mellifera]
gene2111	94.90833311	57.01151327	1.664722223	0.735281468	0.047875	gi|571513290|ref|XP_006565983.1|	PREDICTED: chaoptin-like, partial [Apis mellifera]
gene12883	6920.564154	4169.69429	1.659729388	0.730948036	0.000406	gi|110768640|ref|XP_001119842.1|	PREDICTED: uncharacterized protein LOC724114 isoform X1 [Apis mellifera]
gene11520	105.249448	63.46140389	1.658479667	0.729861325	0.040948	gi|571575360|ref|XP_006563786.1|	PREDICTED: enteropeptidase-like [Apis mellifera]
gene2686	310.5977176	187.6978287	1.654775229	0.726635267	0.005278	gi|755959992|ref|XP_011304587.1|	PREDICTED: 60S ribosomal protein L37a [Fopius arisanus]
gene1627	63.63914685	38.48005969	1.653821417	0.725803458	0.03918	gi|571510150|ref|XP_006571692.1|	PREDICTED: HIG1 domain family member 1B-like [Apis mellifera]
gene3840	113.6148063	68.82044416	1.650887432	0.723241751	0.012887	gi|571525805|ref|XP_006567379.1|	PREDICTED: alkylglycerol monooxygenase-like [Apis mellifera]
gene12219	1591.884701	968.9893872	1.642829862	0.716183076	0.000137	gi|328793032|ref|XP_003251816.1|	PREDICTED: uncharacterized protein LOC100578392 [Apis mellifera]

gene6373	3424.826568	2085.309292	1.642359041	0.715769553	8.86E-05	gi|292494904|ref|NP_001167614.1|	ribosomal protein L35 [Apis mellifera]
gene11062	182.7940141	111.378945	1.64119003	0.714742295	0.008318	gi|328778021|ref|XP_395665.4|	PREDICTED: protein brown [Apis mellifera]
gene5460	162.9473969	100.0187726	1.629168133	0.7041355	0.043331	gi|328791967|ref|XP_392873.4|	PREDICTED: intraflagellar transport protein 74 homolog [Apis mellifera]
gene6780	506.6409439	312.6918118	1.620256511	0.696222231	0.032521	gi|110758964|ref|XP_001121327.1|	PREDICTED: regucalcin-like [Apis mellifera]
gene3264	16162.91477	9983.25126	1.619003103	0.695105751	0.004329	.	.
gene4622	610.2399838	377.7869068	1.6153021	0.691804008	0.015633	gi|66509585|ref|XP_395132.2|	PREDICTED: uncharacterized protein LOC411664 isoform X2 [Apis mellifera]
gene6926	3682.461504	2281.697634	1.613913013	0.690562822	2.32E-05	gi|48142237|ref|XP_397314.1|	PREDICTED: 60S ribosomal protein L31 isoform 2 [Apis mellifera]
gene12255	2214.706449	1372.287447	1.613879405	0.690532779	0.038978	gi|66558893|ref|XP_623595.1|	PREDICTED: probable cytochrome P450 6a14 [Apis mellifera]
gene3735	1085.352191	673.4438002	1.611644788	0.688533804	0.000325	gi|322788733|gb|EFZ14326.1|	hypothetical protein SINV_15101, partial [Solenopsis invicta]
gene6981	70.03160562	43.58984274	1.606603769	0.684014166	0.043748	gi|328789207|ref|XP_395655.4|	PREDICTED: jmjC domain-containing protein 4-like isoform 1 [Apis mellifera]
gene7961	140.6766141	87.6563087	1.604865824	0.682452685	0.015444	gi|571553130|ref|XP_006562937.1|	PREDICTED: uncharacterized protein LOC102656803 [Apis mellifera]
gene1	848.5324	528.8873	1.6043	0.6820094	0.001	gi|66552169|ref|	PREDICTED: 60S ribosomal protein L28 [Apis mellifera]

2807	418	265	72802	14	07	XP_625152.1|	
gene1<br>0089	91.37530<br>037	57.07505<br>397	1.6009<br>67393	0.6789439<br>25	0.025<br>45	gi|571567042|ref<br>|XP_393912.3|	PREDICTED: L-threonine 3-dehydrogenase, mitochondrial-like [Apis mellifera]
gene1<br>1122	270.8088<br>802	169.5725<br>443	1.5970<br>09005	0.6753724<br>47	0.005<br>843	gi|572261912|ref<br>|XP_006609229.<br>1|	PREDICTED: ABC transporter F family member 4-like isoform X1 [Apis dorsata]
gene1<br>0156	181.7887<br>897	113.9685<br>764	1.5950<br>78183	0.6736271<br>4	0.007<br>145	gi|571568097|ref<br>|XP_006560490.<br>1|	PREDICTED: fumarylacetoacetase [Apis mellifera]
gene1<br>0050	208.8176<br>948	131.0258<br>685	1.5937<br>13495	0.6723922<br>96	0.019<br>776	gi|571566849|ref<br>|XP_006558585.<br>1|	PREDICTED: G patch domain-containing protein 4-like [Apis mellifera]
gene1<br>2212	144.3213<br>196	90.58885<br>643	1.5931<br>465	0.6718789<br>38	0.025<br>308	.	.
gene4<br>740	151.8323<br>459	95.35135<br>134	1.5923<br>46031	0.6711538<br>81	0.021<br>231	gi|380025726|ref<br>|XP_003696619.<br>1|	PREDICTED: adenosine 3'-phospho 5'-phosphosulfate transporter 1-like [Apis florea]
gene1<br>1955	4973.884<br>95	3124.167<br>14	1.5920<br>67494	0.6709014<br>99	3.51E<br>-05	gi|571576123|ref<br>|XP_624943.3|	PREDICTED: 40S ribosomal protein S7 [Apis mellifera]
gene7<br>309	712.4011<br>865	447.4915<br>237	1.5919<br>88113	0.6708295<br>64	0.001<br>033	gi|110766380|ref<br>|XP_001122696.<br>1|	PREDICTED: protein takeout-like [Apis mellifera]
gene3<br>723	1213.706<br>297	764.3367<br>455	1.5879<br>20905	0.6671390<br>53	0.002<br>861	gi|571525079|ref<br>|XP_392316.4|	PREDICTED: protein-L-isoaspartate(D-aspartate) O-methyltransferase-like isoform X5 [Apis mellifera]
gene5<br>581	4812.736<br>391	3047.206<br>045	1.5793<br>93162	0.6593703<br>49	0.001<br>138	gi|48105889|ref| XP_393034.1|	PREDICTED: 60S ribosomal protein L7a [Apis mellifera]

gene1769	236.4629791	150.3283505	1.572976609	0.653497217	0.029966	gi|571511216|ref|XP_006559166.1|	PREDICTED: putative leucine-rich repeat-containing protein DDB_G0290503-like isoform X1 [Apis mellifera]
gene12935	374.8107307	238.7255692	1.570048538	0.65080916	0.001416	gi|571578681|ref|XP_001123051.2|	PREDICTED: glutamine synthetase 2 cytoplasmic-like, partial [Apis mellifera]
gene7353	4860.177505	3095.711634	1.569971005	0.650737915	0.000286	gi|571547971|ref|XP_006561561.1|	PREDICTED: 40S ribosomal protein S8 isoform X1 [Apis mellifera]
gene12523	508.7198806	324.2436817	1.56894308	0.649793013	0.002279	gi|110773426|ref|XP_001119845.1|	PREDICTED: stress-associated endoplasmic reticulum protein 2-like [Apis mellifera]
gene10910	1045.05807	667.1882344	1.566361659	0.647417357	0.000782	gi|110762954|ref|XP_001121176.1|	PREDICTED: 60S ribosomal protein L34 isoformX1 [Apis mellifera]
gene6927	289.431639	185.0682429	1.563918447	0.645165283	0.004629	gi|66514688|ref|XP_624939.1|	PREDICTED: protein phosphatase 1 regulatory subunit 14C-like isoform X3 [Apis mellifera]
gene6628	172.4100579	110.3308537	1.562664043	0.644007647	0.023817	gi|328783839|ref|XP_395385.3|	PREDICTED: 2-amino-3-ketobutyrate coenzyme A ligase, mitochondrial-like isoform X3 [Apis mellifera]
gene675	228.6949349	146.694601	1.55898672	0.640608639	0.006562	gi|110765976|ref|XP_623942.2|	PREDICTED: BAG family molecular chaperone regulator 2-like [Apis mellifera]
gene7830	4771.016851	3062.029695	1.558122333	0.639808508	0.000392	gi|572268139|ref|XP_006612127.1|	PREDICTED: 60S acidic ribosomal protein P0-like [Apis dorsata]
gene12536	112.1575481	72.01196702	1.557484856	0.639218135	0.030947	gi|110769776|ref|XP_001123277.	PREDICTED: dolichyl-diphosphooligosaccharide--protein glycosyltransferase subunit DAD1-like [Apis mellifera]

						1|	
gene8 178	3302.244 223	2120.849 01	1.5570 38812	0.6388049 07	0.000 388	gi|297591983|ref |NP_001172073. 1|	60S acidic ribosomal protein P1 [Apis mellifera]
gene5 501	2407.637 324	1549.273 432	1.5540 42866	0.6360262 99	0.000 513	gi|48104663|ref| XP_392962.1|	PREDICTED: receptor of activated protein kinase C 1 isoformX1 [Apis mellifera]
gene5 872	205.6179 247	132.3768 627	1.5532 76914	0.6353150 52	0.014 82	gi|383855926|ref |XP_003703461. 1|	PREDICTED: 60S ribosomal protein L29-1 [Megachile rotundata]
gene6 733	214.1447 796	137.9364 547	1.5524 88645	0.6345827 16	0.011 467	gi|66514532|ref| XP_624335.1|	PREDICTED: guanine nucleotide-binding protein subunit beta-2-like [Apis mellifera]
gene4 916	208.6058 852	134.5900 447	1.5499 35477	0.6322081 58	0.022 409	gi|328780683|ref |XP_001121779. 2|	PREDICTED: UDP-glucuronosyltransferase 2C1-like [Apis mellifera]
gene1 0905	795.9347 489	513.7851 316	1.5491 58782	0.6314850 22	0.001 858	gi|571572991|ref |XP_006565801. 1|	PREDICTED: ATP synthase subunit d, mitochondrial [Apis mellifera]
gene1 1421	4588.458 448	2962.781 73	1.5486 99454	0.6310571 97	0.000 268	gi|66525285|ref| XP_392071.2|	PREDICTED: 60S ribosomal protein L4 isoform 1 [Apis mellifera]
gene1 0316	1107.689 082	715.3997 65	1.5483 49798	0.6307314 38	0.000 873	gi|571568772|ref |XP_006565136. 1|	PREDICTED: uncharacterized protein LOC724946 [Apis mellifera]
gene5 578	538.5641 202	348.0574 701	1.5473 42512	0.6297925 8	0.023 084	gi|66547599|ref| XP_623499.1|	PREDICTED: selection and upkeep of intraepithelial T-cells protein 2-like [Apis mellifera]
gene1 2634	2229.472 419	1445.390 181	1.5424 70987	0.6252433 54	0.001 234	gi|380023818|ref |XP_003695708.	PREDICTED: 60S ribosomal protein L12 [Apis florea]

						1|	
gene159	6613.180983	4288.531006	1.542062066	0.624860833	0.000635	gi|110767080|ref|XP_001120419.1|	PREDICTED: 40S ribosomal protein S6-like [Apis mellifera]
gene8665	100.8168581	65.3967775	1.541618134	0.624445447	0.036965	gi|571557896|ref|XP_006559087.1|	PREDICTED: uncharacterized protein LOC551609 [Apis mellifera]
gene4123	2453.098591	1595.197363	1.537802562	0.620870288	0.000481	gi|572272221|ref|XP_006614024.1|	PREDICTED: 40S ribosomal protein S11-like isoform X1 [Apis dorsata]
gene12761	512.9703758	333.9740527	1.535958772	0.619139492	0.002126	gi|572316891|ref|XP_006624058.1|	PREDICTED: opsin, ultraviolet-sensitive-like [Apis dorsata]
gene315	210.9857635	137.5455267	1.533934025	0.617236433	0.009767	gi|571501317|ref|XP_006562598.1|	PREDICTED: eyes absent homolog 4 isoform X1 [Apis mellifera]
gene10549	6640.047713	4334.924239	1.531756346	0.615186829	0.000668	gi|66524293|ref|XP_393965.2|	PREDICTED: 40S ribosomal protein SA [Apis mellifera]
gene9835	3515.794923	2295.783193	1.531414174	0.614864515	0.030337	gi|571565205|ref|XP_006566903.1|	PREDICTED: cytochrome P450 6AQ1 isoform X1 [Apis mellifera]
gene5558	150.9899175	98.80574639	1.528149151	0.611785361	0.022225	gi|328791922|ref|XP_395661.4|	PREDICTED: transcriptional adapter 1-like isoform 1 [Apis mellifera]
gene795	2379.977355	1558.660928	1.526937201	0.610640729	0.00095	gi|805784468|ref|XP_003703131.2|	PREDICTED: 60S ribosomal protein L9 [Megachile rotundata]

gene3323	134.6853094	88.39353334	1.523700935	0.607579765	0.036614	gi|571522164|ref|XP_623316.3|	PREDICTED: cell division cycle protein 16 homolog isoform X2 [Apis mellifera]
gene1560	109.3595296	71.81670235	1.522758996	0.606687627	0.049548	gi|118150500|ref|NP_001071287.1|	chemosensory protein 6 precursor [Apis mellifera]
gene10647	5327.139446	3504.063774	1.520274684	0.604332014	0.000892	gi|66551967|ref|XP_624896.1|	PREDICTED: 60S ribosomal protein L13 isoform 1 [Apis mellifera]
gene10797	2393.566045	1575.001743	1.519722791	0.603808189	0.001877	gi|110762813|ref|XP_625009.2|	PREDICTED: 60S ribosomal protein L22 isoform 1 [Apis mellifera]
gene3414	3586.784712	2368.188378	1.514569004	0.598907309	0.002158	gi|156554296|ref|XP_001602465.1|	PREDICTED: 40S ribosomal protein S14 [Nasonia vitripennis]
gene387	2204.721783	1459.620292	1.510476248	0.595003498	0.00074	gi|571501696|ref|XP_006563278.1|	PREDICTED: 60S ribosomal protein L10a [Apis mellifera]
gene11506	377.0098466	249.9222387	1.508508601	0.593122922	0.007848	gi|66525117|ref|XP_625185.1|	PREDICTED: prefoldin subunit 6-like isoform X2 [Apis mellifera]
gene203	546.653249	362.5128886	1.507955348	0.5925937 1	0.004989	gi|572272330|ref|XP_006614075.1|	PREDICTED: uncharacterized protein LOC102676956 [Apis dorsata]
gene5557	370.79277	246.3909339	1.504896159	0.589663941	0.006421	gi|66564836|ref|XP_624899.1|	PREDICTED: AP-3 complex subunit mu-1-like isoform X2 [Apis mellifera]
gene7437	368.7736118	245.1387924	1.504346204	0.589136621	0.010134	gi|571549345|ref|XP_006561839.1|	PREDICTED: probable elongation factor 1-delta isoform X1 [Apis mellifera]
gene5	1736.076	1154.644	1.5035	0.5883813	0.008	gi|58585214|ref|	FABP-like protein [Apis mellifera]

01	667	95	5888	65	493	NP_001011636.<br>1|	
gene3<br>500	3250.027<br>054	2162.707<br>953	1.5027<br>58174	0.5876128<br>67	0.001<br>585	gi|571523338|ref<br>|XP_006562063.<br>1|	PREDICTED: ribosome biogenesis protein NSA2 homolog isoform X1 [Apis mellifera]
gene7<br>763	5586.913<br>719	3718.362<br>951	1.5025<br>19736	0.5873839<br>41	0.001<br>188	gi|571551848|ref<br>|XP_006560185.<br>1|	PREDICTED: peroxisomal hydratase-dehydrogenase-epimerase-like [Apis mellifera]
gene7<br>324	2895.001<br>686	1927.235<br>422	1.5021<br>52592	0.5870313<br>73	0.001<br>351	gi|571548748|ref<br>|XP_006561728.<br>1|	PREDICTED: 60S ribosomal protein L35a isoform X1 [Apis mellifera]
gene2<br>92	5213.762<br>025	3472.328<br>928	1.5015<br>1732	0.5864211<br>16	0.000<br>959	gi|350404704|ref<br>|XP_003487192.<br>1|	PREDICTED: 40S ribosomal protein S17 [Bombus impatiens]
gene7<br>079	223.7018<br>804	149.0716<br>845	1.5006<br>32941	0.5855711<br>33	0.017<br>671	gi|328790583|ref<br>|XP_624669.3|	PREDICTED: serine/threonine-protein phosphatase 4 catalytic subunit B-like, partial [Apis mellifera]
gene8<br>922	1257.721<br>632	1889.859<br>54	0.6655<br>1064	-0.5874663<br>63	0.001<br>647	gi|328787292|ref<br>|XP_003250919.<br>1|	PREDICTED: LOW QUALITY PROTEIN: ral guanine nucleotide dissociation stimulator [Apis mellifera]
gene2<br>241	1260.805<br>484	1897.689<br>482	0.6643<br>89773	-0.5898982<br>3	0.007<br>999	gi|572313600|ref<br>|XP_006622495.<br>1|	PREDICTED: retinal-specific ATP-binding cassette transporter-like [Apis dorsata]
gene6<br>631	696.7102<br>089	1048.727<br>575	0.6643<br>386	-0.5900093<br>53	0.017<br>337	gi|571544325|ref<br>|XP_006563880.<br>1|	PREDICTED: plasma membrane calcium-transporting ATPase 3 isoform X3 [Apis mellifera]
gene9	385.8362	581.4370	0.6635	-0.5916341	0.027	gi|571563105|ref	PREDICTED: dynein heavy chain 7, axonemal-like [Apis mellifera]

337	693	111	90831	43	38	|XP_006570553.1|	
gene5589	208.6096514	314.3808929	0.663557029	-0.591707632	0.019658	gi|571536790|ref|XP_006571000.1|	PREDICTED: LON peptidase N-terminal domain and RING finger protein 3-like isoform X1 [Apis mellifera]
gene7736	605.8484711	913.8552149	0.662958925	-0.593008606	0.0061	gi|571551614|ref|XP_006559285.1|	PREDICTED: SNF-related serine/threonine-protein kinase-like [Apis mellifera]
gene2453	2119.28637	3200.208476	0.662233847	-0.594587345	0.013316	gi|571514351|ref|XP_006568803.1|	PREDICTED: uncharacterized protein LOC410793 [Apis mellifera]
gene10937	744.5965592	1124.502932	0.662156174	-0.594756568	0.017533	gi|571573081|ref|XP_006565834.1|	PREDICTED: transcription factor Sp1-like [Apis mellifera]
gene5720	508.9623143	769.4301774	0.661479533	-0.596231577	0.008766	gi|328777755|ref|XP_396693.2|	PREDICTED: carboxylesterase 5A [Apis mellifera]
gene11518	973.7595348	1472.114539	0.661469953	-0.596252471	0.006147	gi|571574799|ref|XP_006563582.1|	PREDICTED: STE20-like serine/threonine-protein kinase-like isoform X1 [Apis mellifera]
gene1601	533.5376994	806.8457359	0.66126358	-0.596702651	0.015182	gi|571510304|ref|XP_006571710.1|	PREDICTED: short spindle protein 4 isoform X1 [Apis mellifera]
gene1228	36762.01204	55614.13429	0.661019227	-0.59723586	0.00289	gi|571507693|ref|XP_006571189.1|	PREDICTED: titin-like isoform X1 [Apis mellifera]
gene7	2027.470	3069.567	0.6605	-0.5983547	0.012	gi|328781112|ref	PREDICTED: leukocyte receptor cluster member 8 homolog isoform X1 [Apis

387	269	713	06774	37	592	|XP_001121422.2|	mellifera]
gene617	3034.557521	4596.369716	0.660207448	-0.59900868	0.003329	gi|380027719|ref|XP_003697566.1|	PREDICTED: 3-phosphoinositide-dependent protein kinase 1 isoform X1 [Apis florea]
gene9638	1123.906313	1702.936543	0.659981323	-0.599502897	0.002223	gi|571564323|ref|XP_006562313.1|	PREDICTED: metalloproteinase inhibitor 3-like isoform X2 [Apis mellifera]
gene4103	743.3420354	1127.512202	0.659276267	-0.601044948	0.005713	gi|571527866|ref|XP_006562261.1|	PREDICTED: putative leucine-rich repeat-containing protein DDB_G0290503-like [Apis mellifera]
gene3287	236.809703	359.7960822	0.658177548	-0.603451282	0.015084	gi|571521962|ref|XP_006558737.1|	PREDICTED: POU domain, class 2, transcription factor 1-like isoform X2 [Apis mellifera]
gene3055	145.267498	220.9723452	0.657401259	-0.605153874	0.037812	gi|861649330|gb|KMQ96764.1|	hypothetical protein RF55_2936, partial [Lasius niger]
gene1317	1113.354915	1694.109271	0.657191914	-0.605613365	0.002624	gi|571508489|ref|XP_006571351.1|	PREDICTED: actin-binding LIM protein 2-like isoform X2 [Apis mellifera]
gene4669	303.3761928	461.6768516	0.657118051	-0.605775522	0.005701	gi|301601654|ref|NP_001013377.2|	venom acid phosphatase Acph-1 precursor [Apis mellifera]
gene11283	279.7410828	425.8461851	0.656906396	-0.606240282	0.022387	gi|328780356|ref|XP_624636.3|	PREDICTED: serine/arginine repetitive matrix protein 2-like isoformX2 [Apis mellifera]
gene1139	2125.234073	3236.130269	0.656720804	-0.606647936	0.005314	gi|571506210|ref|XP_006570777.	PREDICTED: apoptosis 2 inhibitor-like [Apis mellifera]

						1|	
gene9274	1495.522346	2278.895388	0.656248792	-0.607685232	0.002727	gi|66519258|ref|XP_625210.1|	PREDICTED: glycine N-methyltransferase-like [Apis mellifera]
gene5244	329.0128635	502.0608797	0.655324637	-0.609718325	0.022638	gi|571534392|ref|XP_006565398.1|	PREDICTED: uncharacterized protein LOC100577669 isoform X2 [Apis mellifera]
gene151	738.4531787	1128.155878	0.654566619	-0.611388065	0.009483	gi|571500407|ref|XP_006559949.1|	PREDICTED: protein muscleblind-like [Apis mellifera]
gene10984	1126.669035	1722.070033	0.654252739	-0.612080037	0.009733	gi|571573297|ref|XP_006558002.1|	PREDICTED: histone-lysine N-methyltransferase 2C [Apis mellifera]
gene4169	89.01634454	136.1354228	0.65388084	-0.612900346	0.036949	gi|571527928|ref|XP_006564221.1|	PREDICTED: estradiol 17-beta-dehydrogenase 2 [Apis mellifera]
gene9757	247.2242566	378.1922026	0.653700036	-0.613299318	0.02658	gi|571564875|ref|XP_006565275.1|	PREDICTED: myelin regulatory factor-like isoform X1 [Apis mellifera]
gene2671	104.0874874	159.489074	0.652630834	-0.615660944	0.037144	gi|48095402|ref|XP_392288.1|	PREDICTED: TNF receptor-associated factor 4-like isoform X4 [Apis mellifera]
gene8689	549.5118987	842.7640115	0.652035316	-0.616977988	0.030695	gi|571557908|ref|XP_006559090.1|	PREDICTED: anion exchange protein 2-like [Apis mellifera]
gene6347	190.3543911	292.0276264	0.651836929	-0.617417007	0.033644	gi|328777846|ref|XP_395849.4|	PREDICTED: transient receptor potential cation channel trpm isoform X10 [Apis mellifera]
gene8	643.9774	988.2161	0.6516	-0.6178165	0.007	gi|571557888|ref	PREDICTED: serine/threonine-protein kinase N [Apis mellifera]

<table>
<tr><td>590</td><td>264</td><td>718</td><td>56434</td><td>47</td><td>631</td><td>|XP_006559086.1|</td><td></td></tr>
<tr><td>gene2113</td><td>1180.002995</td><td>1811.574578</td><td>0.651368709</td><td>-0.618453678</td><td>0.002875</td><td>gi|328786226|ref|XP_392617.4|</td><td>PREDICTED: neurotrimin [Apis mellifera]</td></tr>
<tr><td>gene11918</td><td>930.8721797</td><td>1433.02981</td><td>0.649583263</td><td>-0.622413635</td><td>0.006096</td><td>gi|571576060|ref|XP_006559482.1|</td><td>PREDICTED: protein sprouty, partial [Apis mellifera]</td></tr>
<tr><td>gene8760</td><td>1301.985088</td><td>2004.762925</td><td>0.649445913</td><td>-0.622718715</td><td>0.00189</td><td>gi|571558282|ref|XP_006563373.1|</td><td>PREDICTED: uncharacterized protein LOC412215 isoform X1 [Apis mellifera]</td></tr>
<tr><td>gene9813</td><td>319.1079968</td><td>491.3766104</td><td>0.649416334</td><td>-0.622784424</td><td>0.008513</td><td>gi|328785548|ref|XP_003250614.1|</td><td>PREDICTED: uncharacterized protein LOC100576266 isoform X1 [Apis mellifera]</td></tr>
<tr><td>gene1342</td><td>991.8351702</td><td>1527.884322</td><td>0.649155931</td><td>-0.623363031</td><td>0.007096</td><td>gi|571508634|ref|XP_006571379.1|</td><td>PREDICTED: LOW QUALITY PROTEIN: endoribonuclease Dicer-like [Apis mellifera]</td></tr>
<tr><td>gene1135</td><td>206.7467424</td><td>318.7626945</td><td>0.6485914</td><td>-0.6246182</td><td>0.00832</td><td>gi|571506988|ref|XP_006570927.1|</td><td>PREDICTED: uncharacterized protein LOC411996 [Apis mellifera]</td></tr>
<tr><td>gene8482</td><td>120.4238029</td><td>185.8702875</td><td>0.647891627</td><td>-0.626175583</td><td>0.029855</td><td>gi|571556504|ref|XP_003249116.2|</td><td>PREDICTED: dentin sialophosphoprotein-like isoform X1 [Apis mellifera]</td></tr>
<tr><td>gene3884</td><td>419.9787278</td><td>648.8468691</td><td>0.647269406</td><td>-0.627561779</td><td>0.008279</td><td>gi|571526129|ref|XP_006569380.1|</td><td>PREDICTED: focal adhesion kinase 1 isoform X1 [Apis mellifera]</td></tr>
<tr><td>gene1</td><td>139.2713</td><td>215.4316</td><td>0.6464</td><td>-0.6293312</td><td>0.018</td><td>gi|328782396|ref</td><td>PREDICTED: uncharacterized protein LOC412825 isoform X2 [Apis mellifera]</td></tr>
</table>

1331	628	036	76007	68	388	|XP_396277.4|	
gene2978	709.999147	1098.63927	0.646253203	-0.629828569	0.027198	gi|328787394|ref|XP_001120785.2|	PREDICTED: zinc finger protein 704-like isoform X1 [Apis mellifera]
gene11974	99.80772077	154.9822329	0.643994598	-0.634879509	0.044001	gi|328780859|ref|XP_623612.3|	PREDICTED: uncharacterized protein LOC551214 [Apis mellifera]
gene1451	992.1353899	1543.807685	0.642654781	-0.637884131	0.004004	gi|820854688|ref|XP_012344633.1|	PREDICTED: sarcoplasmic reticulum histidine-rich calcium-binding protein-like [Apis florea]
gene9992	370.474215	576.9541378	0.642120735	-0.639083509	0.011378	gi|571566328|ref|XP_006557910.1|	PREDICTED: probable serine/threonine-protein kinase CG32666-like isoform X1 [Apis mellifera]
gene4099	421.2642988	656.1754337	0.641999498	-0.639355926	0.003739	gi|571527860|ref|XP_006562260.1|	PREDICTED: putative leucine-rich repeat-containing protein DDB_G0290503-like [Apis mellifera]
gene2765	400.1117635	623.6461057	0.641568607	-0.640324544	0.003744	gi|572300533|ref|XP_006616356.1|	PREDICTED: guanylate cyclase 32E-like [Apis dorsata]
gene3605	373.3388511	582.4810282	0.640945942	-0.641725412	0.034388	gi|571523965|ref|XP_006562759.1|	PREDICTED: rap guanine nucleotide exchange factor 6-like isoform X5 [Apis mellifera]
gene3639	888.7253551	1386.782416	0.640854214	-0.641931895	0.025267	gi|328785371|ref|XP_001121634.2|	PREDICTED: beta-1,3-glucan-binding protein [Apis mellifera]
gene10139	163.6633409	255.4125379	0.640780372	-0.642098139	0.041345	gi|571567534|ref|XP_006560383.	PREDICTED: uncharacterized protein LOC410462 [Apis mellifera]

						1|	
gene1017	230.9547054	361.3424517	0.63915741	-0.645756818	0.0307	gi|571506073|ref|XP_006570707.1|	PREDICTED: myogenic-determination protein [Apis mellifera]
gene6481	231.4456644	362.3720395	0.638696255	-0.646798105	0.020154	gi|571543074|ref|XP_392487.4|	PREDICTED: uncharacterized protein C20orf112 homolog isoform X3 [Apis mellifera]
gene2167	1609.150936	2521.328659	0.638215462	-0.647884533	0.002763	gi|328788856|ref|XP_003251195.1|	PREDICTED: uncharacterized protein LOC410867 [Apis mellifera]
gene8102	14585.81003	22867.27822	0.637846353	-0.64871915	0.023674	gi|571555466|ref|XP_006566278.1|	PREDICTED: loricrin-like isoform X1 [Apis mellifera]
gene768	1630.065966	2562.125116	0.63621638	-0.652410579	0.003778	gi|571503986|ref|XP_006568691.1|	PREDICTED: triple functional domain protein isoform X1 [Apis mellifera]
gene9842	3410.973436	5365.197228	0.635759188	-0.653447687	0.000341	gi|328787572|ref|XP_393878.3|	PREDICTED: IGF-II mRNA-binding protein isoform X4 [Apis mellifera]
gene6686	147.8046081	232.64984	0.635309305	-0.654468946	0.012271	gi|66514147|ref|XP_396290.2|	PREDICTED: alpha-mannosidase 2 isoform X5 [Apis mellifera]
gene1472	2314.129305	3645.182416	0.634845953	-0.655521534	0.00305	gi|48094759|ref|XP_392183.1|	PREDICTED: quinone oxidoreductase-like isoformX1 [Apis mellifera]
gene3047	243.4078469	383.52714	0.634656121	-0.655952995	0.003119	gi|48095262|ref|XP_394396.1|	PREDICTED: putative inorganic phosphate cotransporter-like isoform X2 [Apis mellifera]
gene6910	580.1977958	914.2378647	0.634624552	-0.656024757	0.003506	gi|66555130|ref|XP_394934.2|	PREDICTED: sterol regulatory element-binding protein cleavage-activating protein-like isoform X5 [Apis mellifera]
gene1	196.5258	309.6814	0.6346	-0.6560658	0.022	gi|571568747|ref	PREDICTED: putative polypeptide N-acetylgalactosaminyltransferase 9-like isoform

0269	321	049	06499		169	|XP_006565129.1|	X1 [Apis mellifera]
gene3702	238.6390384	376.5313923	0.633782583	-0.657940082	0.008501	gi|571524982|ref|XP_392312.3|	PREDICTED: signal-induced proliferation-associated 1-like protein 2-like isoform X3 [Apis mellifera]
gene1166	1611.66751	2543.790164	0.633569361	-0.658425525	0.004672	gi|571507165|ref|XP_006571083.1|	PREDICTED: calponin homology domain-containing protein DDB_G0272472-like isoform X1 [Apis mellifera]
gene9071	2739.972881	4326.543743	0.633293697	-0.659053375	0.023743	gi|571560383|ref|XP_006566549.1|	PREDICTED: ataxin-2 homolog isoform X2 [Apis mellifera]
gene11309	222.1247696	350.7890256	0.633214706	-0.659233334	0.00907	gi|66547762|ref|XP_395162.2|	PREDICTED: E3 ubiquitin-protein ligase KCMF1-like isoform X2 [Apis mellifera]
gene11476	152.5791903	241.416078	0.632017518	-0.661963549	0.027074	.	.
gene4884	165.9914729	263.6013762	0.629706397	-0.667248772	0.012946	gi|571532845|ref|XP_006560108.1|	PREDICTED: dynein heavy chain 5, axonemal [Apis mellifera]
gene6337	319.9521158	508.1625498	0.629625532	-0.66743405	0.019576	gi|328777836|ref|XP_397383.3|	PREDICTED: myocyte-specific enhancer factor 2 isoform X6 [Apis mellifera]
gene6461	125.426207	199.3830756	0.629071483	-0.668704132	0.047356	gi|571542912|ref|XP_006559626.1|	PREDICTED: protein SMG9-like [Apis mellifera]
gene2362	285.9674696	456.1506057	0.626914589	-0.673659191	0.009382	gi|66557637|ref|XP_395638.2|	PREDICTED: SH3 domain-containing RING finger protein 3 isoformX2 [Apis mellifera]
gene4360	1484.316847	2371.109793	0.626000893	-0.675763381	0.000762	gi|328788553|ref|XP_392382.4|	PREDICTED: protein similar-like [Apis mellifera]

gene1779	597.7658412	955.5302369	0.625585479	-0.676721069	0.003902	gi|571511127|ref|XP_006559148.1|	PREDICTED: ALK tyrosine kinase receptor-like isoform X1 [Apis mellifera]
gene8110	879.6185029	1406.161808	0.625545722	-0.676812758	0.001491	gi|571555411|ref|XP_006566271.1|	PREDICTED: RNA-binding protein 24-like isoform X3 [Apis mellifera]
gene7438	993.4827362	1589.24913	0.625127123	-0.677778496	0.001275	gi|571549403|ref|XP_006561854.1|	PREDICTED: solute carrier organic anion transporter family member 5A1-like isoform X1 [Apis mellifera]
gene4782	227.0690198	363.822403	0.624120499	-0.680103497	0.022237	gi|571531755|ref|XP_006570259.1|	PREDICTED: lutropin-choriogonadotropic hormone receptor-like isoform X2 [Apis mellifera]
gene2015	182.5369452	292.6248582	0.623791657	-0.680863838	0.01076	gi|571512920|ref|XP_006565932.1|	PREDICTED: dihydropyrimidinase-like isoform X2 [Apis mellifera]
gene3659	12445.81341	19966.66673	0.623329551	-0.681932984	0.000657	gi|571524355|ref|XP_006566009.1|	PREDICTED: microtubule-associated protein futsch [Apis mellifera]
gene1062	263.0838868	422.1214878	0.62324211	-0.682135381	0.011145	gi|571506745|ref|XP_006570883.1|	PREDICTED: shootin-1-like isoform X1 [Apis mellifera]
gene10306	309.6483173	496.8460343	0.623227914	-0.682168244	0.034398	gi|48105753|ref|XP_395996.1|	PREDICTED: long-chain-fatty-acid--CoA ligase 6-like isoform X5 [Apis mellifera]
gene1492	472.6985722	759.2866228	0.622556171	-0.683724084	0.011794	gi|571509864|ref|XP_006571637.1|	PREDICTED: serine protease snake isoform X2 [Apis mellifera]

gene10793	412.7201068	663.2559784	0.622263681	-0.68440205	0.012998	gi|328785091|ref|XP_392015.3|	PREDICTED: chloride channel protein 2-like isoform X5 [Apis mellifera]
gene2647	748.1476116	1204.593328	0.621078993	-0.687151323	0.028392	gi|328778864|ref|XP_397465.4|	PREDICTED: ATP-binding cassette sub-family A member 2-like isoform X2 [Apis mellifera]
gene5083	753.2765479	1212.884917	0.621061848	-0.687191149	0.000612	gi|820849087|ref|XP_012342290.1|	PREDICTED: protein fem-1 homolog CG6966 isoform X1 [Apis florea]
gene6694	321.2426928	517.4803033	0.620782454	-0.687840313	0.008905	gi|571544009|ref|XP_006563828.1|	PREDICTED: forkhead box protein I1-like isoform X1 [Apis mellifera]
gene8608	93.95164715	151.3934962	0.62057915	-0.688312869	0.013545	gi|571557367|ref|XP_006559002.1|	PREDICTED: homeobox protein caupolican isoform X1 [Apis mellifera]
gene8965	535.4672792	864.532306	0.619372203	-0.691121457	0.001139	gi|328786814|ref|XP_391908.4|	PREDICTED: RB1-inducible coiled-coil protein 1 isoform X3 [Apis mellifera]
gene8175	578.8175925	935.6506948	0.618625728	-0.692861259	0.00585	gi|571555016|ref|XP_006566205.1|	PREDICTED: serine/threonine-protein kinase mig-15 isoform X1 [Apis mellifera]
gene6564	792.0705929	1280.72885	0.618452995	-0.693264147	0.001607	gi|571543610|ref|XP_006561489.1|	PREDICTED: protein unc-13 homolog D isoform X1 [Apis mellifera]
gene11549	265.2728322	429.0484764	0.618281725	-0.693663731	0.015935	gi|571574890|ref|XP_006563617.1|	PREDICTED: atrial natriuretic peptide receptor 1-like isoform X1 [Apis mellifera]
gene658	371.725921	601.3951999	0.6181059	-0.69407406	0.002041	gi|328788793|ref|XP_396802.3|	PREDICTED: ubiquitin carboxyl-terminal hydrolase 31-like isoform 1 [Apis mellifera]

<table>
<tr><td>gene3755</td><td>185.5576605</td><td>300.2353322</td><td>0.618040719</td><td>-0.694226204</td><td>0.044007</td><td>gi|571524747|ref|XP_006567170.1|</td><td>PREDICTED: NF-kappa-B inhibitor cactus 1 isoform X1 [Apis mellifera]</td></tr>
<tr><td>gene1637</td><td>101.0548568</td><td>163.728917</td><td>0.617208363</td><td>-0.696170485</td><td>0.035418</td><td>gi|328792845|ref|XP_393414.4|</td><td>PREDICTED: leishmanolysin-like peptidase [Apis mellifera]</td></tr>
<tr><td>gene5650</td><td>551.5399004</td><td>896.6670204</td><td>0.615100018</td><td>-0.701107077</td><td>0.020003</td><td>gi|571537270|ref|XP_006557373.1|</td><td>PREDICTED: dentin sialophosphoprotein-like isoform X1 [Apis mellifera]</td></tr>
<tr><td>gene1161</td><td>1046.258023</td><td>1701.806552</td><td>0.61479257</td><td>-0.701828366</td><td>0.040891</td><td>gi|328792136|ref|XP_397003.4|</td><td>PREDICTED: uncharacterized protein R02F2.2-like isoform X5 [Apis mellifera]</td></tr>
<tr><td>gene12102</td><td>215.5989798</td><td>351.0136041</td><td>0.614218302</td><td>-0.703176594</td><td>0.010742</td><td>gi|571576575|ref|XP_006563214.1|</td><td>PREDICTED: uncharacterized protein LOC724469 isoform X1 [Apis mellifera]</td></tr>
<tr><td>gene3261</td><td>51.35972251</td><td>83.6297591</td><td>0.614132135</td><td>-0.703379</td><td>0.037528</td><td>gi|571521847|ref|XP_006558249.1|</td><td>PREDICTED: uncharacterized protein LOC100576135 [Apis mellifera]</td></tr>
<tr><td>gene9471</td><td>320.1715281</td><td>521.5858046</td><td>0.613842488</td><td>-0.704059587</td><td>0.023819</td><td>gi|571563474|ref|XP_006558394.1|</td><td>PREDICTED: uncharacterized protein LOC725246 [Apis mellifera]</td></tr>
<tr><td>gene9913</td><td>405.4579153</td><td>660.861095</td><td>0.613529709</td><td>-0.70479489</td><td>0.016983</td><td>gi|571565878|ref|XP_006567026.1|</td><td>PREDICTED: tolkin isoform X1 [Apis mellifera]</td></tr>
<tr><td>gene9160</td><td>258.9864</td><td>422.3264113</td><td>0.613237517</td><td>-0.705482132</td><td>0.013645</td><td>.</td><td>.</td></tr>
<tr><td>gene9918</td><td>50.16886892</td><td>81.85240702</td><td>0.612918676</td><td>-0.70623243</td><td>0.038787</td><td>gi|571565909|ref|XP_006567030.</td><td>PREDICTED: protein trachealess-like isoform X1 [Apis mellifera]</td></tr>
</table>

						1|	
gene11752	447.3820239	730.8174613	0.612166577	-0.708003815	0.004821	gi|571575649|ref|XP_006557820.1|	PREDICTED: mitogen-activated protein kinase-binding protein 1-like isoform X1 [Apis mellifera]
gene11177	173.8495484	284.3633121	0.611364199	-0.709896024	0.034172	gi|328792187|ref|XP_003251692.1|	PREDICTED: uncharacterized protein LOC100577522 isoform X1 [Apis mellifera]
gene8414	220.4216443	360.9995773	0.610586987	-0.711731252	0.018656	gi|328789808|ref|XP_393661.3|	PREDICTED: disks large homolog 5-like isoform X6 [Apis mellifera]
gene9910	82.97990821	135.9211379	0.610500394	-0.71193587	0.023396	gi|571566042|ref|XP_006567059.1|	PREDICTED: uncharacterized protein LOC726758 [Apis mellifera]
gene3817	655.1878775	1074.566329	0.609723067	-0.713773969	0.028089	gi|572307746|ref|XP_006619721.1|	PREDICTED: CCAAT/enhancer-binding protein-like [Apis dorsata]
gene11522	8045.384023	13203.68287	0.609328784	-0.714707203	0.025278	gi|328783370|ref|XP_001121121.2|	PREDICTED: trypsin-1-like isoform X1 [Apis mellifera]
gene605	47.49572922	77.98080358	0.609069502	-0.715321228	0.043962	gi|571502866|ref|XP_006565377.1|	PREDICTED: golgin subfamily A member 6-like protein 2-like isoform X1 [Apis mellifera]
gene7184	74.17648672	121.7934879	0.609034917	-0.715403153	0.033584	gi|820866416|ref|XP_012349637.1|	PREDICTED: uncharacterized protein LOC105737031 [Apis florea]
gene3596	4591.144077	7542.208999	0.608726711	-0.716133422	0.000404	gi|328782416|ref|XP_001120871.	PREDICTED: uncharacterized protein LOC724971 isoform X1 [Apis mellifera]

						2|	
gene10161	252.5057419	414.868155	0.608640935	-0.716336727	0.005515	gi|66522653|ref|XP_391978.2|	PREDICTED: LIM domain-containing protein jub-like [Apis mellifera]
gene2559	470.3899273	773.146682	0.608409682	-0.716884982	0.001302	gi|328777221|ref|XP_397251.4|	PREDICTED: formin-binding protein 1 homolog isoform X8 [Apis mellifera]
gene5499	1277.131669	2100.269493	0.608079903	-0.717667186	0.016907	gi|571537009|ref|XP_006571028.1|	PREDICTED: CD109 antigen [Apis mellifera]
gene11597	180.3892695	296.8062949	0.607767667	-0.718408168	0.013864	gi|571575051|ref|XP_006563676.1|	PREDICTED: disintegrin and metalloproteinase domain-containing protein 10 isoform X1 [Apis mellifera]
gene3385	616.0233045	1013.825983	0.607622328	-0.718753209	0.001636	gi|572299676|ref|XP_006615958.1|	PREDICTED: uncharacterized protein LOC102675370 [Apis dorsata]
gene7317	998.5994946	1643.693259	0.607533972	-0.718963011	0.000477	gi|571548729|ref|XP_006561725.1|	PREDICTED: microtubule-associated protein futsch isoform X3 [Apis mellifera]
gene36	193.6319435	318.944573	0.607102174	-0.719988756	0.013724	gi|571499372|ref|XP_006557431.1|	PREDICTED: LOW QUALITY PROTEIN: probable E3 ubiquitin-protein ligase MYCBP2-like [Apis mellifera]
gene10696	71.06284036	117.1301334	0.606699901	-0.720945019	0.042748	gi|571571591|ref|XP_006563142.1|	PREDICTED: flocculation protein FLO11-like, partial [Apis mellifera]
gene4197	338.2446956	558.0638059	0.606103983	-0.722362771	0.013235	gi|571528328|ref|XP_006564287.1|	PREDICTED: ral GTPase-activating protein subunit alpha-1-like [Apis mellifera]

gene9303	1357.214374	2240.471674	0.605771718	-0.723153871	0.002075	gi|571562868|ref|XP_006570507.1|	PREDICTED: histone deacetylase 5 isoform X1 [Apis mellifera]
gene4612	132.0186994	218.1163086	0.605267438	-0.724355354	0.01224	gi|571531460|ref|XP_006570008.1|	PREDICTED: phosphoinositide 3-kinase adapter protein 1-like isoform X1 [Apis mellifera]
gene1444	269.1543242	444.7627554	0.605163811	-0.724602378	0.003659	gi|571509220|ref|XP_006571490.1|	PREDICTED: glutamate receptor ionotropic, kainate 2-like isoform X1 [Apis mellifera]
gene6289	66.64707816	110.3271855	0.604085728	-0.727174792	0.015377	gi|328777523|ref|XP_001121647.2|	PREDICTED: uncharacterized protein LOC725844 [Apis mellifera]
gene10038	854.64363	1415.04013	0.603971302	-0.727448093	0.00161	gi|571566790|ref|XP_006558573.1|	PREDICTED: transmembrane protein 132C-like isoform X1 [Apis mellifera]
gene2727	1938.121704	3213.24344	0.603166781	-0.729371119	0.000605	gi|571518233|ref|XP_006560559.1|	PREDICTED: uncharacterized protein LOC413858 isoform X1 [Apis mellifera]
gene12472	168.1755813	279.0546382	0.602661839	-0.730579381	0.012083	gi|328793368|ref|XP_001122855.2|	PREDICTED: uncharacterized protein C9orf85 homolog [Apis mellifera]
gene6707	1362.07538	2264.995041	0.6013591	-0.733701344	0.004769	gi|571544563|ref|XP_006563931.1|	PREDICTED: basement membrane-specific heparan sulfate proteoglycan core protein-like [Apis mellifera]
gene5936	78357.23001	130709.5025	0.599476155	-0.738225725	0.005507	gi|571539411|ref|XP_006564376.	PREDICTED: titin isoform X14 [Apis mellifera]

						1|	
gene10768	186.7431317	312.0300478	0.598478041	-0.740629782	0.021968	gi|328785145|ref|XP_003250547.1|	PREDICTED: uncharacterized protein LOC100578776 isoform X1 [Apis mellifera]
gene5703	409.473053	685.4657536	0.597364713	-0.743316077	0.002266	gi|328776788|ref|XP_396348.4|	PREDICTED: octopamine receptor beta-2R isoformX5 [Apis mellifera]
gene3054	557.4711172	933.8435121	0.59696417	-0.744283752	0.007447	gi|571520595|ref|XP_006568504.1|	PREDICTED: uncharacterized protein LOC725508 isoform X1 [Apis mellifera]
gene1528	658.1839158	1104.107168	0.596123216	-0.746317535	0.007504	gi|328792704|ref|XP_003251765.1|	PREDICTED: G-protein coupled receptor Mth2-like [Apis mellifera]
gene4097	3975.288999	6675.929408	0.595466003	-0.747908953	0.001414	gi|571527472|ref|XP_006562194.1|	PREDICTED: muscle M-line assembly protein unc-89 isoform X1 [Apis mellifera]
gene7931	766.5217484	1289.277169	0.594536044	-0.750163817	0.017306	gi|571552976|ref|XP_006562907.1|	PREDICTED: microtubule-associated protein futsch-like isoform X1 [Apis mellifera]
gene7351	48.61301972	81.81063941	0.594213907	-0.750945723	0.033365	gi|571548873|ref|XP_006561744.1|	PREDICTED: uncharacterized protein LOC102654791 isoform X1 [Apis mellifera]
gene1983	119.8910223	201.7896673	0.59413856	-0.751128671	0.01249	gi|571512714|ref|XP_006565907.1|	PREDICTED: uncharacterized protein LOC100578356 isoform X1 [Apis mellifera]
gene7569	419.9354931	707.6226704	0.593445505	-0.752812538	0.006059	gi|328788058|ref|XP_003251054.	PREDICTED: protein sprint-like [Apis mellifera]

						1|	
gene8 280	150.7346 158	254.4962 887	0.5922 86106	-0.7556338 52	0.019 855	gi|328786663|ref |XP_001121883. 2|	PREDICTED: trichohyalin isoform X1 [Apis mellifera]
gene2 350	594.0028 259	1002.967 681	0.5922 45231	-0.7557334 18	0.017 572	gi|571514834|ref |XP_006568889. 1|	PREDICTED: bestrophin-2-like isoform X1 [Apis mellifera]
gene9 824	574.5077 208	970.4640 229	0.5919 92807	-0.7563484 49	0.009 484	gi|820860434|ref |XP_012347067. 1|	PREDICTED: hairy/enhancer-of-split related with YRPW motif protein 1 isoform X1 [Apis florea]
gene4 751	428.2637 754	723.8136 737	0.5916 76824	-0.7571187 1	0.000 898	gi|571532211|ref |XP_006570323. 1|	PREDICTED: myosin-VIIa [Apis mellifera]
gene1 87	112.2280 872	189.8431 325	0.5911 62218	-0.7583740 28	0.011 205	gi|571500415|ref |XP_006559951. 1|	PREDICTED: translation initiation factor eIF-2B subunit epsilon-like [Apis mellifera]
gene7 791	114.3814 26	193.6603 448	0.5906 29053	-0.7596757 72	0.007 605	gi|571552109|ref |XP_003249836. 2|	PREDICTED: O-glucosyltransferase rumi homolog isoform X1 [Apis mellifera]
gene7 087	242.5186 845	412.5713 616	0.5878 22391	-0.7665477 79	0.006 393	gi|571547082|ref |XP_006569898. 1|	PREDICTED: dentin sialophosphoprotein [Apis mellifera]
gene2 789	211.6413 505	360.3941 357	0.5872 49707	-0.7679540 07	0.002 026	gi|571518556|ref |XP_006560971. 1|	PREDICTED: ATP-binding cassette sub-family C member Sur isoform X1 [Apis mellifera]
gene1	1504.814	2563.186	0.5870	-0.7683532	9.20E	gi|571574872|ref	PREDICTED: DE-cadherin-like isoform X1 [Apis mellifera]

1545	321	982	87221	41	-05	|XP_006563610.<br>1|	
gene1<br>772	602.3701<br>865	1026.229<br>275	0.5869<br>74277	-0.7686308<br>14	0.007<br>414	gi|328778092|ref<br>|XP_003249448.<br>1|	PREDICTED: putative transcription factor capicua-like isoform X1 [Apis mellifera]
gene5<br>535	50.92504<br>592	86.80138<br>106	0.5866<br>84743	-0.7693426<br>21	0.025<br>136	.	.
gene6<br>228	55.18665<br>674	94.06699<br>037	0.5866<br>73992	-0.7693690<br>58	0.034<br>56	gi|328790219|ref<br>|XP_003251395.<br>1|	PREDICTED: uncharacterized protein LOC100578532 [Apis mellifera]
gene7<br>489	74.25034<br>434	126.7310<br>416	0.5858<br>89167	-0.7713003<br>21	0.007<br>608	gi|328781401|ref<br>|XP_001120510.<br>2|	PREDICTED: neprilysin-2 isoform X1 [Apis mellifera]
gene2<br>216	2694.368<br>596	4603.078<br>505	0.5853<br>4057	-0.7726518<br>19	0.029<br>946	gi|571515814|ref<br>|XP_006569086.<br>1|	PREDICTED: ryanodine receptor 44F isoform X1 [Apis mellifera]
gene1<br>0713	187.6079<br>999	321.8287<br>202	0.5829<br>43622	-0.7785717<br>31	0.012<br>493	gi|571571585|ref<br>|XP_006563140.<br>1|	PREDICTED: uncharacterized protein LOC408462 [Apis mellifera]
gene4<br>165	1290.149<br>797	2220.898<br>284	0.5809<br>13501	-0.7836047<br>35	0.000<br>319	gi|571527923|ref<br>|XP_006564220.<br>1|	PREDICTED: ribosome-releasing factor 2, mitochondrial-like [Apis mellifera]
gene2<br>90	123.3381<br>322	212.3288<br>265	0.5808<br>82654	-0.7836813<br>45	0.023<br>111	gi|571500821|ref<br>|XP_006561977.<br>1|	PREDICTED: uncharacterized protein LOC102653658 [Apis mellifera]
gene5	67.13570	115.6059	0.5807	-0.7840632	0.015	.	.

120	96	356	28915	25	785		
gene8393	46.84174775	80.87903085	0.579158124	-0.787970803	0.034656	.	.
gene10034	44.24382777	76.39871617	0.579117425	-0.788072189	0.047631	.	.
gene3079	117.6367821	203.9695793	0.576736896	-0.794014775	0.027589	gi|571520772|ref|XP_006568534.1|	PREDICTED: succinate dehydrogenase [ubiquinone] flavoprotein subunit, mitochondrial-like [Apis mellifera]
gene2321	1166.830139	2027.456931	0.575514143	-0.797076712	0.000216	gi|571515056|ref|XP_006568926.1|	PREDICTED: uncharacterized protein LOC725893 isoform X2 [Apis mellifera]
gene12892	183.9954124	320.0585348	0.574880506	-0.798665985	0.006475	gi|571578616|ref|XP_006572452.1|	PREDICTED: filamin-A-like [Apis mellifera]
gene10195	110.9936395	193.8242462	0.572650954	-0.804272049	0.007103	gi|328779553|ref|XP_001121446.2|	PREDICTED: lachesin-like [Apis mellifera]
gene2328	2411.604985	4214.758495	0.57218106	-0.80545635	0.000144	gi|571514985|ref|XP_006568907.1|	PREDICTED: microtubule-associated protein futsch-like isoform X1 [Apis mellifera]
gene2730	95.24785639	166.6885839	0.571411996	-0.807396772	0.012745	gi|571518206|ref|XP_006560552.1|	PREDICTED: dual oxidase isoform X1 [Apis mellifera]
gene3256	138.8088097	243.0170749	0.571189534	-0.80795855	0.002468	gi|328776907|ref|XP_624136.3|	PREDICTED: transcriptional activator cubitus interruptus isoform X2 [Apis mellifera]
gene9	1026.303	1797.468	0.5709	-0.8085087	0.000	gi|571562942|ref	PREDICTED: uncharacterized protein LOC408333 isoform X1 [Apis mellifera]

296	46	067	71735	67	46	|XP_006570521.1|	
gene7413	53.33314305	93.42692334	0.570854109	-0.808806007	0.01958	gi|571549265|ref|XP_006561821.1|	PREDICTED: uncharacterized protein LOC100578091 [Apis mellifera]
gene7676	131.9770873	231.3469165	0.570472645	-0.809770387	0.014535	gi|571550740|ref|XP_006567539.1|	PREDICTED: transcription factor Sp8-like [Apis mellifera]
gene11165	77.65491069	136.3422605	0.569558627	-0.812083742	0.018024	gi|328779954|ref|XP_394109.3|	PREDICTED: G protein-coupled receptor kinase 2 isoform X2 [Apis mellifera]
gene5940	13158.27386	23235.23383	0.566306926	-0.820343919	0.015201	gi|571539415|ref|XP_006564377.1|	PREDICTED: titin-like [Apis mellifera]
gene11835	121.6574978	215.6097719	0.564248534	-0.825597329	0.01819	gi|328778440|ref|XP_623903.2|	PREDICTED: homeotic protein Sex combs reduced [Apis mellifera]
gene1856	979.8056918	1743.035347	0.562126117	-0.831034249	0.008518	gi|328780582|ref|XP_393610.4|	PREDICTED: rho guanine nucleotide exchange factor 12 isoform X4 [Apis mellifera]
gene11699	245.7112455	437.8011755	0.561239346	-0.833311941	0.013403	gi|66554761|ref|XP_397331.2|	PREDICTED: ankyrin repeat and SOCS box protein 16-like isoform X2 [Apis mellifera]
gene3967	1112.705355	1983.131479	0.561085014	-0.833708713	0.026652	gi|571526651|ref|XP_006557955.1|	PREDICTED: uncharacterized protein LOC102655788 [Apis mellifera]
gene3829	647.9128849	1156.454344	0.560258075	-0.835836556	0.000248	gi|334724440|ref|NP_001229316.1|	filamin-like [Apis mellifera]
gene3	1191.702	2128.499	0.5598	-0.8368121	0.001	gi|571521734|ref	PREDICTED: zinc finger protein 1-like [Apis mellifera]

235	967	676	79327	83	162	|XP_006557404.1|	
gene4934	308.6458936	551.9530034	0.55918872	-0.838592835	0.0005	gi|571533018|ref|XP_006561305.1|	PREDICTED: probable phospholipid-transporting ATPase ID-like isoform X1 [Apis mellifera]
gene8463	2340.853831	4195.204192	0.557983289	-0.841706181	0.009457	gi|571556170|ref|XP_006569704.1|	PREDICTED: abaecin isoform X1 [Apis mellifera]
gene3140	121.2763199	217.4227238	0.557790454	-0.84220485	0.00917	gi|328789915|ref|XP_001120679.2|	PREDICTED: G-protein coupled receptor Mth2-like [Apis mellifera]
gene3491	346.3923036	621.1596051	0.557654266	-0.842557137	0.001539	gi|110755844|ref|XP_396884.3|	PREDICTED: protein kibra-like isoformX2 [Apis mellifera]
gene10281	451.855521	811.0068634	0.557153757	-0.843852574	0.000865	gi|328785317|ref|XP_001120437.2|	PREDICTED: integrin alpha-PS2 isoform X1 [Apis mellifera]
gene7848	241.62712	433.8862796	0.556890437	-0.844534575	0.000728	gi|571552520|ref|XP_006562428.1|	PREDICTED: gamma-tubulin complex component 5-like, partial [Apis mellifera]
gene8361	1624.112131	2916.713903	0.556829427	-0.844692639	0.000147	gi|571555508|ref|XP_006566284.1|	PREDICTED: LOW QUALITY PROTEIN: kinase D-interacting substrate of 220 kDa-like [Apis mellifera]
gene5038	437.1798776	786.1936402	0.556071501	-0.846657695	0.000375	gi|328782190|ref|XP_394861.4|	PREDICTED: nose resistant to fluoxetine protein 6-like [Apis mellifera]
gene11810	465.4494826	837.9655602	0.555451805	-0.848266358	0.003422	gi|571575481|ref|XP_006557753.	PREDICTED: growth factor receptor-bound protein 14-like isoform X2 [Apis mellifera]

						1|	
gene7921	920.7560859	1662.295165	0.553906493	-0.852285644	0.003657	gi|328782724|ref|XP_393711.3|	PREDICTED: protein kinase DC2 isoform X2 [Apis mellifera]
gene3655	120.1303151	217.5027851	0.552316215	-0.856433611	0.019218	gi|571524339|ref|XP_006566006.1|	PREDICTED: ETS-like protein pointed, isoform P1-like isoform X1 [Apis mellifera]
gene293	214.7277095	389.1927684	0.551725846	-0.857976528	0.007644	gi|571500707|ref|XP_006561972.1|	PREDICTED: furin-like isoform X1 [Apis mellifera]
gene7729	38.81224861	70.40892821	0.551240441	-0.859246362	0.037266	gi|66518356|ref|XP_395932.2|	PREDICTED: knirps-related protein isoform X3 [Apis mellifera]
gene4104	1330.572987	2420.827967	0.549635499	-0.86345291	8.94E-05	gi|571527532|ref|XP_006562205.1|	PREDICTED: dentin sialophosphoprotein-like [Apis mellifera]
gene4810	1774.559912	3232.714016	0.548938107	-0.865284602	7.58E-05	gi|58585174|ref|NP_001011615.1|	hymenoptaecin preproprotein [Apis mellifera]
gene245	27.81387623	50.6956174	0.54864459	-0.866056215	0.037025	gi|571501010|ref|XP_006562006.1|	PREDICTED: autophagy-related protein 23-like isoform X1 [Apis mellifera]
gene8442	26.27644332	48.09953033	0.546293137	-0.872252795	0.026813	gi|328790437|ref|XP_623893.3|	PREDICTED: Bardet-Biedl syndrome 5 protein homolog [Apis mellifera]
gene5125	464.9121149	851.6184251	0.545915989	-0.873249142	0.009325	gi|571533916|ref|XP_006564143.1|	PREDICTED: sushi, von Willebrand factor type A, EGF and pentraxin domain-containing protein 1-like isoform X1 [Apis mellifera]
gene1	92.87560	170.4184	0.5449	-0.8757099	0.011	gi|328792499|ref	PREDICTED: major facilitator superfamily domain-containing protein 12-like

435	616	576	85605	72	766	|XP_624057.2|	isoform X2 [Apis mellifera]
gene7497	269.5589499	495.0417992	0.544517554	-0.876949535	0.001779	gi|571549689|ref|XP_006561911.1|	PREDICTED: organic solute transporter alpha-like protein-like [Apis mellifera]
gene3479	191.0674834	351.1611622	0.544101979	-0.878051019	0.000407	gi|571523199|ref|XP_006562043.1|	PREDICTED: kinetochore-associated protein 1-like [Apis mellifera]
gene2437	5390.454677	9907.457278	0.544080537	-0.878107874	0.000939	gi|571514423|ref|XP_006568818.1|	PREDICTED: LOW QUALITY PROTEIN: twitchin [Apis mellifera]
gene7226	352.1656826	649.4281216	0.542270454	-0.882915528	0.006065	gi|571548223|ref|XP_006561630.1|	PREDICTED: la-related protein 4-like isoform X1 [Apis mellifera]
gene2936	210.1127632	388.4259669	0.540933874	-0.88647585	0.014878	gi|571519626|ref|XP_003250376.2|	PREDICTED: uncharacterized protein LOC100577178 isoform X1 [Apis mellifera]
gene3179	591.5339784	1095.332288	0.540049796	-0.888835656	0.000232	gi|572297383|ref|XP_006614882.1|	PREDICTED: protein crumbs-like isoform X1 [Apis dorsata]
gene7000	40.52292013	75.2339133	0.538625712	-0.892644994	0.020759	gi|571546304|ref|XP_006568595.1|	PREDICTED: cAMP-specific 3',5'-cyclic phosphodiesterase, isoforms N/G isoform X9 [Apis mellifera]
gene12577	122.0396975	226.8014939	0.53809036	-0.894079634	0.000831	gi|328793514|ref|XP_395995.3|	PREDICTED: organic cation transporter protein-like isoform 1 [Apis mellifera]
gene4978	385.6083453	716.7141701	0.538022494	-0.894261603	0.043769	gi|571533289|ref|XP_006561360.	PREDICTED: uncharacterized protein LOC725964 isoform X1 [Apis mellifera]

						1|	
gene13340	85.37982222	158.808002	0.53762922	-0.895316544	0.022628	gi|328794317|ref|XP_001123227.2|	PREDICTED: short spindle protein 4-like [Apis mellifera]
gene4364	364.0765407	677.6822877	0.537237799	-0.896367281	0.000117	gi|571529190|ref|XP_006567731.1|	PREDICTED: centrosomal and chromosomal factor-like isoform X1 [Apis mellifera]
gene11853	44.49606611	82.9826606	0.536209201	-0.899132121	0.037561	.	.
gene11391	68.98188973	128.7376318	0.535833142	-0.900144279	0.024847	gi|571575347|ref|XP_006563782.1|	PREDICTED: neurofilament medium polypeptide-like [Apis mellifera]
gene1209	8231.385857	15384.16287	0.535055819	-0.902238689	0.000565	gi|328792196|ref|XP_001121241.2|	PREDICTED: probable serine/threonine-protein kinase clkA-like [Apis mellifera]
gene7811	23.10989267	43.29933462	0.533723968	-0.905834296	0.035218	gi|572305324|ref|XP_006618592.1|	PREDICTED: uncharacterized protein LOC102678905 [Apis dorsata]
gene3021	377.4088751	707.7993651	0.533214487	-0.907212118	0.010986	gi|571520122|ref|XP_006568040.1|	PREDICTED: guanine nucleotide-releasing factor 2-like isoform X1 [Apis mellifera]
gene1537	571.5571532	1076.599089	0.530891359	-0.913511436	0.000687	gi|66501273|ref|XP_392808.2|	PREDICTED: tribbles homolog 2 isoform X2 [Apis mellifera]
gene10957	74.49084601	140.3371073	0.530799355	-0.913761478	0.007081	gi|571573151|ref|XP_006565859.1|	PREDICTED: facilitated trehalose transporter Tret1-like isoform X1 [Apis mellifera]

gene10825	255.6422321	483.1018137	0.529168438	-0.91820108	0.000672	gi|571572421|ref|XP_006565003.1|	PREDICTED: protein ccsmst1-like isoform X2 [Apis mellifera]
gene7264	35.40543977	67.05525795	0.528003931	-0.921379425	0.045847	gi|571548069|ref|XP_006561588.1|	PREDICTED: sodium channel protein paralytic isoform X16 [Apis mellifera]
gene1592	437.2792416	829.8480737	0.526938913	-0.924292373	0.031172	gi|328792769|ref|XP_003251772.1|	PREDICTED: uncharacterized protein LOC100578606 isoform X1 [Apis mellifera]
gene6984	88.52572791	168.4609885	0.525496904	-0.92824583	0.028175	gi|66535525|ref|XP_624087.1|	PREDICTED: thioredoxin domain-containing protein 9-like [Apis mellifera]
gene4765	20923.09459	39855.4985	0.524973853	-0.929682527	1.97E-06	gi|572315326|ref|XP_006623317.1|	PREDICTED: uncharacterized protein LOC102673964 [Apis dorsata]
gene1829	664.3570676	1265.994895	0.524770732	-0.930240836	0.000111	gi|571511699|ref|XP_006561153.1|	PREDICTED: pleckstrin homology-like domain family B member 2-like isoform X1 [Apis mellifera]
gene3362	346.0527325	659.7843173	0.524493722	-0.93100259	0.013257	gi|571523080|ref|XP_006560680.1|	PREDICTED: protein held out wings [Apis mellifera]
gene4164	1181.265858	2259.040136	0.522906096	-0.935376206	0.011032	gi|571527908|ref|XP_006564216.1|	PREDICTED: transcription factor kayak isoform X1 [Apis mellifera]
gene8785	191.6641266	367.0591328	0.522161443	-0.937432163	0.001683	gi|328784384|ref|XP_003250447.1|	PREDICTED: transcriptional regulator Myc-B-like [Apis mellifera]

gene11296	143.952646	275.8066402	0.521933213	-0.938062883	0.001711	gi|571574152|ref|XP_006561080.1|	PREDICTED: protein TANC2-like isoform X1 [Apis mellifera]
gene10294	74.50743025	142.9572212	0.521186895	-0.940127286	0.003518	gi|110762343|ref|XP_001120949.1|	PREDICTED: protein odd-skipped-like [Apis mellifera]
gene7453	75.87599362	145.6163151	0.521067942	-0.940456598	0.012307	gi|571549438|ref|XP_006561864.1|	PREDICTED: DBF4-type zinc finger-containing protein 2 homolog isoform X1 [Apis mellifera]
gene8492	2108.704491	4047.854954	0.520943689	-0.940800662	3.87E-05	gi|571556561|ref|XP_006557505.1|	PREDICTED: protein-methionine sulfoxide oxidase Mical isoform X1 [Apis mellifera]
gene8982	440.4994557	847.7738227	0.519595491	-0.944539183	0.015187	.	.
gene4446	57.61611763	110.9036011	0.5195153	-0.944761856	0.035495	gi|328788288|ref|XP_003251097.1|	PREDICTED: solute carrier family 52, riboflavin transporter, member 3-A-like [Apis mellifera]
gene1316	58.4744253	113.1183084	0.516931575	-0.951954768	0.007694	gi|328792339|ref|XP_396231.3|	PREDICTED: chondroitin sulfate proteoglycan 4 isoform X3 [Apis mellifera]
gene1204	111.3118639	216.0035464	0.515324242	-0.956447633	0.007917	gi|571507384|ref|XP_006571131.1|	PREDICTED: tropomyosin-2-like [Apis mellifera]
gene7954	128.1357518	248.7649421	0.51508766	-0.957110118	0.000369	gi|572299973|ref|XP_006616097.1|	PREDICTED: G-protein coupled receptor moody-like isoform X1 [Apis dorsata]
gene7	259.1346	503.2459	0.5149	-0.9575616	0.018	gi|571552117|ref	PREDICTED: matrix metalloproteinase-14-like isoform X1 [Apis mellifera]

792	618	475	26475	47	441	|XP_006561295.1|	
gene3541	82.22652874	160.1168054	0.513540903	-0.961448906	0.004823	gi|571523594|ref|XP_006562689.1|	PREDICTED: PHD finger protein rhinoceros-like isoform X1 [Apis mellifera]
gene7062	1599.908367	3124.904822	0.511986271	-0.96582297	0.00048	gi|110759161|ref|XP_394764.3|	PREDICTED: uncharacterized protein LOC411290 isoform X4 [Apis mellifera]
gene1219	54.71316557	106.9464721	0.511593926	-0.966928959	0.039829	gi|328792207|ref|XP_623471.3|	PREDICTED: diacylglycerol kinase 1 isoform X4 [Apis mellifera]
gene9639	33.25978011	65.06106237	0.511208685	-0.968015749	0.020281	.	.
gene12547	191.7600221	376.9126195	0.508765194	-0.974928119	0.006026	gi|571577791|ref|XP_006572178.1|	PREDICTED: uncharacterized protein LOC552030 isoform X1 [Apis mellifera]
gene7816	418.2899358	822.8447861	0.508346097	-0.976117033	5.75E-05	gi|571552186|ref|XP_006562089.1|	PREDICTED: uncharacterized protein LOC412101 isoform X1 [Apis mellifera]
gene2242	202.862371	399.4236025	0.507887791	-0.977418302	0.000773	.	.
gene6240	24.09454014	47.47878843	0.507480097	-0.978576854	0.025873	gi|571540842|ref|XP_006569464.1|	PREDICTED: uncharacterized protein LOC102656235 [Apis mellifera]
gene7256	633.1860671	1253.845826	0.504995155	-0.985658548	2.40E-05	gi|571548368|ref|XP_006561662.1|	PREDICTED: uncharacterized protein LOC100576640 isoform X3 [Apis mellifera]
gene7	27.14715	53.77944	0.5047	-0.9862536	0.024	gi|571550528|ref	PREDICTED: dynein heavy chain 10, axonemal-like [Apis mellifera]

635	86	306	86905	11	726	|XP_006567505.1|	
gene11798	296.540331	588.8148421	0.503622378	-0.989585705	0.000267	gi|571575747|ref|XP_006557850.1|	PREDICTED: microtubule-associated protein futsch-like [Apis mellifera]
gene4200	93.82113071	186.3316695	0.503516825	-0.989888107	0.006872	gi|571528133|ref|XP_006564252.1|	PREDICTED: homeobox protein SIX2-like isoform X1 [Apis mellifera]
gene7405	53.39629754	106.200216	0.502788973	-0.991975086	0.007054	gi|571549180|ref|XP_006561803.1|	PREDICTED: uncharacterized protein LOC411336 isoform X1 [Apis mellifera]
gene5111	178.9338348	356.2924304	0.502210599	-0.993635619	0.001876	gi|571533996|ref|XP_006564155.1|	PREDICTED: probable serine/threonine-protein kinase dyrk2-like isoform X2 [Apis mellifera]
gene10175	1919.455095	3834.484238	0.500577125	-0.99833573	0.002087	gi|571567823|ref|XP_006560440.1|	PREDICTED: piezo-type mechanosensitive ion channel component 2 isoform X1 [Apis mellifera]
gene12075	1812.873106	3623.007821	0.500377917	-0.998909974	0.000286	gi|328782977|ref|XP_392097.4|	PREDICTED: collagen alpha-1(IV) chain-like isoform 1 [Apis mellifera]
gene4431	2687.497961	5413.60442	0.496434123	-1.010325812	0.000344	gi|571529759|ref|XP_006567837.1|	PREDICTED: dual specificity protein phosphatase 10 [Apis mellifera]
gene11110	222.8969045	449.6028539	0.495763989	-1.012274612	0.000393	gi|328779092|ref|XP_624412.2|	PREDICTED: putative amino acid permease F13H10.3-like [Apis mellifera]
gene3277	667.4635441	1347.672757	0.49527123	-1.013709277	0.00133	gi|572268404|ref|XP_006612252.	PREDICTED: vesicular glutamate transporter 2-like isoform X1 [Apis dorsata]

						1|	
gene10310	270.4031593	550.8279852	0.490903089	-1.026489851	0.000182	gi|571568775|ref|XP_006565137.1|	PREDICTED: talin-1-like [Apis mellifera]
gene11284	25.13949354	51.2355525	0.490665023	-1.027189663	0.032538	.	.
gene9887	116.8886776	238.4525218	0.490196861	-1.02856685	0.01251	gi|571565676|ref|XP_006566994.1|	PREDICTED: putative fatty acyl-CoA reductase CG5065-like [Apis mellifera]
gene4038	144.5028094	295.1621575	0.489570921	-1.030410225	0.005257	gi|328780431|ref|XP_396579.2|	PREDICTED: putative ferric-chelate reductase 1 homolog isoformX1 [Apis mellifera]
gene2768	566.8317017	1161.85199	0.487869114	-1.035433941	0.001104	gi|571518921|ref|XP_006561030.1|	PREDICTED: LOW QUALITY PROTEIN: uncharacterized protein LOC551170 [Apis mellifera]
gene6632	30.42797002	62.43042601	0.487390075	-1.036851222	0.02106	.	.
gene5706	18.70612763	38.52910469	0.485506419	-1.042437726	0.035403	.	.
gene3912	19.61234743	40.46336872	0.484693886	-1.04485421	0.030864	.	.
gene3890	156.3884642	322.6821988	0.484651663	-1.044979893	6.30E-05	gi|328790075|ref|XP_001120790.2|	PREDICTED: laccase-1-like [Apis mellifera]
gene12968	25.41525625	52.58473349	0.483320054	-1.048949237	0.034378	gi|571578735|ref|XP_006572484.1|	PREDICTED: uncharacterized protein LOC102655303 isoform X1 [Apis mellifera]

gene4877	22.2972238	46.14229002	0.483227508	-1.049225513	0.026667	gi|571532839|ref|XP_006560106.1|	PREDICTED: epidermal growth factor receptor-like, partial [Apis mellifera]
gene143	102.1782889	212.0880153	0.481773045	-1.053574418	0.024064	gi|571499603|ref|XP_006558614.1|	PREDICTED: uncharacterized protein LOC102656689 isoform X1 [Apis mellifera]
gene10626	1486.847807	3104.263423	0.478969599	-1.061994007	0.000576	gi|571571446|ref|XP_006563114.1|	PREDICTED: protein Malvolio isoform X1 [Apis mellifera]
gene11412	24.68536712	51.81603494	0.476404016	-1.069742521	0.023582	gi|94158832|ref|NP_001035315.1|	odorant binding protein 9 precursor [Apis mellifera]
gene8912	57.81279874	121.530099	0.475707658	-1.071852845	0.002858	gi|571559390|ref|XP_006566381.1|	PREDICTED: protein apterous isoform X1 [Apis mellifera]
gene10238	18.6039321	39.19115654	0.474697195	-1.074920569	0.019753	gi|48097975|ref|XP_393944.1|	PREDICTED: protein escargot [Apis mellifera]
gene44	14.51937393	30.76668337	0.471918723	-1.083389684	0.036384	.	.
gene1203	22.11475152	47.02613292	0.470265151	-1.088453668	0.017434	.	.
gene12060	88.13765559	187.5772372	0.469873941	-1.089654338	0.011911	gi|571576451|ref|XP_006563160.1|	PREDICTED: putative uncharacterized protein DDB_G0282133-like isoform X1 [Apis mellifera]
gene5670	22.48889572	47.903808	0.469459458	-1.090927523	0.024834	gi|110757922|ref|XP_001122422.	PREDICTED: uncharacterized protein LOC726699 isoform X1 [Apis mellifera]

						1|	
gene9 765	43.17633 249	92.05866 312	0.4690 08902	-1.0923127 9	0.005 313	gi|571564846|ref |XP_006565269. 1|	PREDICTED: uncharacterized protein LOC102654968 [Apis mellifera]
gene1 246	1152.300 995	2464.357 319	0.4675 86817	-1.0966938 38	0.000 335	gi|571508040|ref |XP_003251700. 2|	PREDICTED: uncharacterized protein LOC100576169 [Apis mellifera]
gene3 488	98.75870 984	212.5422 38	0.4646 54512	-1.1057696 78	0.002 537	gi|571523231|ref |XP_006562047. 1|	PREDICTED: target of Myb protein 1-like isoform X1 [Apis mellifera]
gene5 937	78.09882 095	168.3762 846	0.4638 35041	-1.1083162 79	0.049 596	gi|571539358|ref |XP_006564362. 1|	PREDICTED: uncharacterized protein LOC102655685 isoform X1 [Apis mellifera]
gene6 609	11.62572 04	25.09526 828	0.4632 63444	-1.1100952 52	0.047 049	gi|571544547|ref |XP_006563926. 1|	PREDICTED: uncharacterized protein LOC413106 isoform X1 [Apis mellifera]
gene1 1968	136.4073 412	294.7759 828	0.4627 49169	-1.1116976 95	0.000 352	gi|571576379|ref |XP_396342.5|	PREDICTED: A disintegrin and metalloproteinase with thrombospondin motifs 3-like [Apis mellifera]
gene2 464	266.2319 138	578.9697 424	0.4598 37353	-1.1208044 33	5.56E -05	gi|328789675|ref |XP_001122118. 2|	PREDICTED: bone morphogenetic protein 2-B isoform X1 [Apis mellifera]
gene1 469	28.56497 918	62.16860 434	0.4594 7596	-1.1219387 13	0.013 205	gi|572269672|ref |XP_006612850. 1|	PREDICTED: uncharacterized protein LOC102673414 [Apis dorsata]
gene4 778	538.8726 037	1174.725 653	0.4587 22088	-1.1243077 2	0.000 253	gi|110757136|ref |XP_625175.2|	PREDICTED: ETS homologous factor-like [Apis mellifera]

gene5776	193.0432995	421.8704516	0.457589051	-1.127875563	0.001873	gi|571538195|ref|XP_006559876.1|	PREDICTED: calcium release-activated calcium channel protein 1-like [Apis mellifera]
gene10110	115.2733891	251.9724688	0.457484064	-1.128206605	5.94E-05	gi|571567206|ref|XP_006560128.1|	PREDICTED: uncharacterized protein PF11_0207-like isoform X1 [Apis mellifera]
gene6235	147.0143961	322.0994285	0.45642551	-1.131548666	3.31E-06	gi|571540827|ref|XP_394662.5|	PREDICTED: L-lactate dehydrogenase-like isoform X2 [Apis mellifera]
gene4562	315.0313134	694.1185326	0.453858093	-1.139686812	1.83E-05	gi|571530638|ref|XP_006567976.1|	PREDICTED: uncharacterized protein LOC552512 isoform X1 [Apis mellifera]
gene10057	89.85052176	198.0327334	0.453715506	-1.140140131	0.003738	gi|571566871|ref|XP_006558588.1|	PREDICTED: uncharacterized protein LOC102654964 isoform X1 [Apis mellifera]
gene8011	20.54973671	45.2935302	0.45370137	-1.140185078	0.019103	gi|328784632|ref|XP_003250475.1|	PREDICTED: pair-rule protein odd-paired-like [Apis mellifera]
gene1229	6117.113171	13512.03818	0.4527158	-1.143322438	6.76E-06	gi|571507718|ref|XP_006571196.1|	PREDICTED: nesprin-1-like [Apis mellifera]
gene7605	31.93808152	71.44282877	0.44704391	-1.16151155	0.042063	gi|571550280|ref|XP_006567453.1|	PREDICTED: peroxiredoxin 1-like [Apis mellifera]
gene12207	44.56437651	99.96281422	0.445809543	-1.165500596	0.036532	gi|571576963|ref|XP_006571904.1|	PREDICTED: EKC/KEOPS complex subunit TPRKB-like [Apis mellifera]

gene6156	22.63864399	50.86836345	0.445043686	-1.167981134	0.019068	gi|380018207|ref|XP_003693026.1|	PREDICTED: GTP-binding protein REM 1 [Apis florea]
gene3255	741.7174058	1670.296402	0.444063344	-1.171162608	0.007496	gi|571521844|ref|XP_006558248.1|	PREDICTED: intracellular protein transport protein USO1-like [Apis mellifera]
gene8080	16.31159921	36.91915252	0.441819438	-1.178471203	0.037615	.	.
gene7880	1031.128389	2345.292742	0.439658713	-1.185544034	0.00011	gi|571552621|ref|XP_006562848.1|	PREDICTED: mucin-2-like [Apis mellifera]
gene11598	15.44117847	35.3861101	0.436362698	-1.196400316	0.025822	.	.
gene3014	11.96588059	27.51965981	0.434812082	-1.201536065	0.045689	.	.
gene9598	26.1384147	60.21932788	0.434053577	-1.204054962	0.037328	gi|571563998|ref|XP_006561270.1|	PREDICTED: protein penguin [Apis mellifera]
gene7059	100.8806665	233.7943605	0.431493156	-1.212590418	0.004964	gi|571546747|ref|XP_006569833.1|	PREDICTED: dynein beta chain, ciliary-like [Apis mellifera]
gene8418	16.9548265	39.39281786	0.430404003	-1.216236599	0.013774	.	.
gene10783	1103.660831	2576.0249	0.428435622	-1.222849657	0.000468	gi|571572211|ref|XP_006564974.1|	PREDICTED: probable cation-transporting ATPase 13A3-like isoform X1 [Apis mellifera]

gene11710	23.18321227	54.15541765	0.428086667	-1.224025191	0.006229	gi|571575429|ref|XP_006557360.1|	PREDICTED: uncharacterized protein LOC102655415 [Apis mellifera]
gene3045	72.25783656	169.2231722	0.426997294	-1.227701169	0.001679	gi|571520736|ref|XP_006568529.1|	PREDICTED: protein tramtrack, alpha isoform-like isoform X7 [Apis mellifera]
gene4905	308.66384	724.8615703	0.425824533	-1.231669023	0.001088	gi|571532896|ref|XP_006560793.1|	PREDICTED: uncharacterized protein LOC102653609 [Apis mellifera]
gene618	29.54960964	69.80424923	0.423321072	-1.240175789	0.005363	.	.
gene4647	74.77944377	177.1245188	0.422185727	-1.244050288	0.000812	gi|571531318|ref|XP_006569990.1|	PREDICTED: cytochrome b5-like [Apis mellifera]
gene10231	1651.657061	3925.735726	0.420725483	-1.24904889	5.85E-07	gi|571568232|ref|XP_006562813.1|	PREDICTED: uncharacterized protein LOC413596 isoform X1 [Apis mellifera]
gene8480	178.8879748	427.8545885	0.418104608	-1.258064153	0.003556	gi|571556476|ref|XP_006557488.1|	PREDICTED: uncharacterized protein LOC100577693 [Apis mellifera]
gene5984	35.8017973	85.87472005	0.416907296	-1.262201476	0.003819	gi|571540045|ref|XP_006565578.1|	PREDICTED: discoidin domain-containing receptor 2-like [Apis mellifera]
gene2691	1367.423015	3283.304882	0.416477624	-1.263689109	0.000206	gi|571517921|ref|XP_394430.5|	PREDICTED: LOW QUALITY PROTEIN: sterile alpha and TIR motif-containing protein 1 [Apis mellifera]
gene1	221.9638	540.4771	0.4106	-1.2839092	2.78E	gi|328792883|ref	PREDICTED: uncharacterized protein LOC100577920 [Apis mellifera]

659	056	699	81187	35	-06	|XP_003251794.1|	
gene6514	247.8474535	604.1695905	0.410228282	-1.285501135	0.006187	gi|571543280|ref|XP_006559685.1|	PREDICTED: spondin-1-like isoform X1 [Apis mellifera]
gene11908	20.95938544	51.90638989	0.403792009	-1.308315736	0.005155	gi|571575979|ref|XP_006559456.1|	PREDICTED: cytosolic carboxypeptidase NnaD-like isoform X1 [Apis mellifera]
gene7583	25.38256627	62.87708347	0.40368549	-1.308696362	0.020234	gi|571550230|ref|XP_394770.5|	PREDICTED: protein fork head-like isoform 1 [Apis mellifera]
gene8076	27.32298528	67.76251681	0.403216801	-1.31037234	0.006192	gi|571553830|ref|XP_006565605.1|	PREDICTED: paired box pox-meso protein isoform X1 [Apis mellifera]
gene8626	128.6299188	322.4101005	0.398963676	-1.325670695	0.00155	gi|571557263|ref|XP_006558991.1|	PREDICTED: yellow-e3 isoform X1 [Apis mellifera]
gene5985	464.6782132	1164.947414	0.398883424	-1.325960923	9.84E-05	gi|328785804|ref|XP_003250660.1|	PREDICTED: uncharacterized protein LOC100578816 [Apis mellifera]
gene12931	86.3321253	217.4337801	0.397050197	-1.332606682	0.001154	gi|571539407|ref|XP_006564375.1|	PREDICTED: titin isoform X13 [Apis mellifera]
gene12730	1281.379704	3243.389993	0.395074199	-1.339804463	0.001478	gi|571578283|ref|XP_006572347.1|	PREDICTED: laminin subunit alpha [Apis mellifera]
gene7	12.32697	31.35438	0.3931	-1.3468481	0.023	gi|571549449|ref	PREDICTED: putative uncharacterized protein DDB_G0271606-like [Apis

452	708	413	50031	28	757	|XP_006561867.1|	mellifera]
gene5912	522.6240462	1339.171374	0.390259272	-1.357495184	0.000838	gi|66512834|ref|XP_395069.2|	PREDICTED: uridine phosphorylase 2-like isoform X2 [Apis mellifera]
gene10235	112.0288382	287.6034814	0.38952532	-1.360210984	8.49E-05	gi|571579119|ref|XP_003252014.2|	PREDICTED: uncharacterized protein LOC100578648, partial [Apis mellifera]
gene10209	14.917286	38.77830284	0.38468125	-1.378264584	0.013166	gi|571568011|ref|XP_006560472.1|	PREDICTED: neuroendocrine convertase 1-like [Apis mellifera]
gene6695	46.15792488	122.6017594	0.376486643	-1.409329411	0.000564	.	.
gene6179	411.0725185	1093.118204	0.376054956	-1.410984583	9.30E-06	gi|66512130|ref|XP_623621.1|	PREDICTED: probable cytochrome P450 305a1 [Apis mellifera]
gene1826	23.9133619	64.47366658	0.370901225	-1.430893063	0.018669	.	.
gene10410	15.0223301	41.38212495	0.363014952	-1.461899123	0.03495	gi|571569216|ref|XP_006558826.1|	PREDICTED: dynein intermediate chain 3, ciliary-like [Apis mellifera]
gene12187	11.04152201	30.830165	0.358140218	-1.481403558	0.027203	gi|328792988|ref|XP_001122709.2|	PREDICTED: endocuticle structural glycoprotein SgAbd-8-like isoform X1 [Apis mellifera]
gene7444	62.74017873	175.2635379	0.35797622	-1.482064342	0.012542	gi|571549427|ref|XP_006561861.1|	PREDICTED: hydrocephalus-inducing protein-like [Apis mellifera]
gene9	146.1462	410.1990	0.3562	-1.4889113	2.56E	gi|328791431|ref	PREDICTED: single Ig IL-1-related receptor-like [Apis mellifera]

381	518	559	81297	42	-06	|XP_003251562.<br>1|	
gene3<br>198	341.8965<br>932	961.0077<br>082	0.3557<br>68835	-1.4909879<br>56	0.000<br>497	gi|571521398|ref<br>|XP_006569925.<br>1|	PREDICTED: LOW QUALITY PROTEIN: formin-like protein CG32138-like [Apis mellifera]
gene5<br>32	13.55184<br>871	38.42967<br>631	0.3526<br>40199	-1.5037311<br>5	0.013<br>275	gi|571502582|ref<br>|XP_006565052.<br>1|	PREDICTED: glucose dehydrogenase [FAD, quinone] isoform X1 [Apis mellifera]
gene1<br>0630	31.67407<br>249	90.26973<br>802	0.3508<br>82513	-1.5109400<br>47	0.000<br>715	gi|571571370|ref<br>|XP_006563100.<br>1|	PREDICTED: zinc finger protein 423 isoform X1 [Apis mellifera]
gene6<br>153	13.16469<br>083	38.05631<br>945	0.3459<br>26538	-1.5314624	0.029<br>13	gi|571540063|ref<br>|XP_006568339.<br>1|	PREDICTED: uncharacterized protein LOC100576948 isoform X1 [Apis mellifera]
gene1<br>657	15.16134<br>121	44.70687<br>994	0.3391<br>27696	-1.5600994<br>81	0.023<br>624	gi|571510690|ref<br>|XP_006571789.<br>1|	PREDICTED: uncharacterized protein LOC102655202 [Apis mellifera]
gene1<br>1355	32.67047<br>365	96.74080<br>887	0.3377<br>11396	-1.5661372<br>3	7.90E<br>-05	.	.
gene1<br>3195	97.29592<br>46	288.6430<br>16	0.3370<br>80474	-1.5688350<br>37	0.002<br>167	gi|571579119|ref<br>|XP_003252014.<br>2|	PREDICTED: uncharacterized protein LOC100578648, partial [Apis mellifera]
gene1<br>0056	39.12225<br>584	118.7470<br>751	0.3294<br>5869	-1.6018305<br>13	0.001<br>056	gi|571566904|ref<br>|XP_006558594.<br>1|	PREDICTED: uncharacterized protein LOC100577569 [Apis mellifera]
gene1	20.18070	62.18972	0.3245	-1.6236999	0.000	gi|571578992|ref	PREDICTED: A disintegrin and metalloproteinase with thrombospondin motifs

3120	058	55	02165	9	533	|XP_623804.2|	14-like isoform 1 [Apis mellifera]
gene2834	126.1246183	393.6529704	0.320395444	-1.642074462	0.010328	gi|571518900|ref|XP_006561027.1|	PREDICTED: sterile alpha and TIR motif-containing protein 1-like, partial [Apis mellifera]
gene7174	16.2119407	51.11779043	0.317148698	-1.656768673	0.006201	gi|48132776|ref|XP_396702.1|	PREDICTED: uncharacterized protein LOC413256 isoformX1 [Apis mellifera]
gene7467	10.91426458	34.58878417	0.315543458	-1.66408938	0.004085	.	.
gene8868	11.8215009	37.83324908	0.312463275	-1.678241462	0.048231	gi|571559117|ref|XP_006566323.1|	PREDICTED: uncharacterized protein LOC413109 [Apis mellifera]
gene7443	1603.344513	5158.180867	0.310835264	-1.685777908	7.49E-05	gi|571549367|ref|XP_006561846.1|	PREDICTED: fibroin heavy chain isoform X2 [Apis mellifera]
gene1880	13.21835114	43.09203928	0.306746939	-1.704879148	0.003037	gi|571512020|ref|XP_006561218.1|	PREDICTED: uncharacterized protein LOC725506 isoform X3 [Apis mellifera]
gene1363	655.4129007	2145.746971	0.305447431	-1.711003987	1.66E-12	gi|571508732|ref|XP_001120032.3|	PREDICTED: kynurenine/alpha-aminoadipate aminotransferase, mitochondrial-like [Apis mellifera]
gene6906	10.05559751	33.44306053	0.300678148	-1.73370807	0.002316	gi|19570323|dbj|BAB86288.1|	mariner transposase [Apis cerana]
gene10169	17.99085777	64.55634557	0.278684576	-1.843294939	0.000567	.	.
gene280	13.24310149	49.00560397	0.270236471	-1.8877057	0.015052	gi|571500862|ref|XP_006561982.	PREDICTED: chaperone protein dnaJ 1, mitochondrial-like [Apis mellifera]

						1|	
gene10019	56.14144579	246.8273148	0.227452322	-2.136363934	1.61E-11	gi|571566714|ref|XP_006558560.1|	PREDICTED: cyclin-dependent kinase 6 isoform X1 [Apis mellifera]
gene1658	12.2822559	59.08787112	0.207864248	-2.266286454	6.51E-05	gi|571510683|ref|XP_006571787.1|	PREDICTED: uncharacterized protein LOC100577882 isoform X1 [Apis mellifera]
gene4432	17.21091291	86.88849089	0.198080468	-2.335841469	0.02508	gi|571529755|ref|XP_006567836.1|	PREDICTED: uncharacterized protein LOC102653871 [Apis mellifera]
gene4929	13.41665165	70.86385448	0.189329973	-2.401025272	6.97E-05	gi|328780665|ref|XP_003249840.1|	PREDICTED: uncharacterized protein LOC100577819 [Apis mellifera]

Table S2. 22 down-regulated genes that related to the function of muscle Go term enriched in imidacloprid treatment groups

Gene	Putative function	NR:Seq-id	NR:Description
gene6235	somatic muscle development	gi|571540827|ref|XP_394662.5|	PREDICTED: L-lactate dehydrogenase-like isoform X2 [Apis mellifera]
gene10281	muscle attachment	gi|328785317|ref|XP_001120437.2|	PREDICTED: integrin alpha-PS2 isoform X1 [Apis mellifera]
gene151	muscle attachment	gi|571500407|ref|XP_006559949.1|	PREDICTED: protein muscleblind-like [Apis mellifera]
gene3021	muscle attachment	gi|571520122|ref|XP_006568040.1|	PREDICTED: guanine nucleotide-releasing factor 2-like isoform X1 [Apis mellifera]
gene3362	muscle attachment	gi|571523080|ref|XP_006560680.1|	PREDICTED: protein held out wings [Apis mellifera]
gene4877	muscle attachment	gi|571532839|ref|XP_006560106.1|	PREDICTED: epidermal growth factor receptor-like, partial [Apis mellifera]
gene13340	microtube related	gi|328794317|ref|XP_001123227.2|	PREDICTED: short spindle protein 4-like [Apis mellifera]
gene1601	microtube	gi|571510304|ref|XP_006571710.1|	PREDICTED: short spindle protein 4 isoform X1 [Apis mellifera]
gene10161	actin binding	gi|66522653|ref|XP_391978.2|	PREDICTED: LIM domain-containing protein jub-like [Apis mellifera]
gene10310	actin binding	gi|571568775|ref|XP_006565137.1|	PREDICTED: talin-1-like [Apis mellifera]
gene1229	actin binding	gi|571507718|ref|XP_006571196.1|	PREDICTED: nesprin-1-like [Apis mellifera]
gene12892	actin binding	gi|571578616|ref|XP_006572452.1|	PREDICTED: filamin-A-like [Apis mellifera]
gene1317	actin binding	gi|571508489|ref|XP_006571351.1|	PREDICTED: actin-binding LIM protein 2-like isoform X2 [Apis mellifera]
gene3198	actin binding	gi|571521398|ref|XP_006569925.1|	PREDICTED: formin-like protein CG32138-like [Apis mellifera]
gene3829	actin binding	gi|334724440|ref|NP_001229316.1|	PREDICTED:filamin-like [Apis mellifera]
gene3884	actin binding	gi|571526129|ref|XP_006569380.1|	PREDICTED: focal adhesion kinase 1 isoform X1 [Apis mellifera]
gene8492	actin binding	gi|571556561|ref|XP_006557505.1|	PREDICTED: protein-methionine sulfoxide oxidase Mical isoform X1 [Apis mellifera]
gene1228	titin-like	gi|571507693|ref|XP_006571189.1|	PREDICTED: titin-like isoform X1 [Apis mellifera]
gene12931	titin-like	gi|571539407|ref|XP_006564375.1|	PREDICTED: titin isoform X13 [Apis mellifera]
gene5936	titin-like	gi|571539411|ref|XP_006564376.1|	PREDICTED: titin isoform X14 [Apis mellifera]
gene7087		gi|571547082|ref|XP_006569898.1|	PREDICTED: dentin sialophosphoprotein [Apis mellifera]
gene8280		gi|328786663|ref|XP_001121883.2|	PREDICTED: trichohyalin isoform X1 [Apis mellifera]

Table S3. 28 overexpressed ribosomal protein genes Go term enriched in imidacloprid treatment groups

Gene	NR:Seq-id	NR:Description
gene5792	gi|307173970|gb|EFN64700.1|	40S ribosomal protein S15 [Camponotus floridanus]
gene11955	gi|571576123|ref|XP_624943.3|	40S ribosomal protein S7 [Apis mellifera]
gene10549	gi|66524293|ref|XP_393965.2|	40S ribosomal protein SA [Apis mellifera]
gene4123	gi|572272221|ref|XP_006614024.1|	40S ribosomal protein S11-like isoform X1 [Apis dorsata]
gene3414	gi|156554296|ref|XP_001602465.1|	40S ribosomal protein S14 [Nasonia vitripennis]
gene292	gi|350404704|ref|XP_003487192.1|	40S ribosomal protein S17 [Bombus impatiens]
gene13055	gi|759079256|ref|XP_011349442.1|	40S ribosomal protein S19-like isoform X1 [Cerapachys biroi]
gene4229	gi|645015869|ref|XP_001601386.2|	40S ribosomal protein S29 [Nasonia vitripennis]
gene159	gi|110767080|ref|XP_001120419.1|	40S ribosomal protein S6-like [Apis mellifera]
gene7353	gi|571547971|ref|XP_006561561.1|	40S ribosomal protein S8 isoform X1 [Apis mellifera]
gene10910	gi|110762954|ref|XP_001121176.1|	60S ribosomal protein L34 isoformX1 [Apis mellifera]
gene7830	gi|572268139|ref|XP_006612127.1|	60S acidic ribosomal protein P0-like [Apis dorsata]
gene387	gi|571501696|ref|XP_006563278.1|	60S ribosomal protein L10a [Apis mellifera]
gene12634	gi|380023818|ref|XP_003695708.1|	60S ribosomal protein L12 [Apis florea]
gene12807	gi|66552169|ref|XP_625152.1|	60S ribosomal protein L28 [Apis mellifera]
gene5872	gi|383855926|ref|XP_003703461.1|	60S ribosomal protein L29-1 [Megachile rotundata]
gene6926	gi|48142237|ref|XP_397314.1|	60S ribosomal protein L31 isoform 2 [Apis mellifera]
gene8822	gi|380029575|ref|XP_003698444.1|	60S ribosomal protein L32 [Apis florea]
gene7324	gi|571548748|ref|XP_006561728.1|	60S ribosomal protein L35a isoform X1 [Apis mellifera]
gene2686	gi|755959992|ref|XP_011304587.1|	60S ribosomal protein L37a [Fopius arisanus]
gene11421	gi|66525285|ref|XP_392071.2|	60S ribosomal protein L4 isoform 1 [Apis mellifera]
gene795	gi|805784468|ref|XP_003703131.2|	60S ribosomal protein L9 [Megachile rotundata]
gene8178	gi|297591983|ref|NP_001172073.1|	60S acidic ribosomal protein P1 [Apis mellifera]

gene10647	gi|66551967|ref|XP_624896.1|	60S ribosomal protein L13 isoform 1 [Apis mellifera]
gene10797	gi|110762813|ref|XP_625009.2|	60S ribosomal protein L22 isoform 1 [Apis mellifera]
gene11923	gi|66526748|ref|XP_624696.1|	60S ribosomal protein L27a isoform X2 [Apis mellifera]
gene3735	gi|322788733|gb|EFZ14326.1|	hypothetical protein SINV_15101, partial [Solenopsis invicta]
gene6373	gi|292494904|ref|NP_001167614.1|	ribosomal protein L35 [Apis mellifera]

Table S4. Sequences of primers for the genes tested

Gene Name	Gene ID-F(R)	Primers	Product length (bp)
AADAT	gene1363-F	GCTCAATCGTATTCTCTGTCCT	195
	gene1363-R	CTTTCACCTTTCTTTACCTTCC	
Cdk6	gene10019-F	GTTGCCTGAGAACACGATTG	192
	gene10019-R	TTCTGCCCGCTGTACTTCAT	
RpS19	gene13055-F	CTACATTCGCAGCCCAATCG	117
	gene13055-R	GCCTTACGTGCAACACCACC	
Syne1	gene1229-F	ATTGAGAAACTTCCGTAATCGC	240
	gene1229-R	TGAACAGTAACATCCTGAGCCA	
RpL31	gene6926-F	AGTGCTATGAGCGAGGTTGTG	142
	gene6926-R	CATCTGGAGTTCCCATTTGTT	
RpS7	gene11955-F	CTGGAATTGGAAATGAATAGCG	128
	gene11955-R	GCCTTTAACTTTGGCATGGGTA	
Bmp7	gene2464-F	CAGAGGAAGGGATCAAACAGAT	263
	gene2464-R	AAGCACATGAAGTAAGGGACTG	
RPL35	gene6373-F	CCTTTTTCCGTTGAAGAGGTAT	138
	gene6373-R	ACTTTTGCCACTCGAAGATTTG	
SP28	gene3689-F	CTGTCTACCGTTCCAACACTCG	126

	gene3689-R	AGCACGCTCACCGTAACTTTCT	
mapf	gene2328-F	TTCTCTCTCCCTAACACCTTGC	145
	gene2328-R	AGATAAATTCGATCATCCCGTC	
β-actin	actin-F	TTGGTATGCCAACACTGTCCTTT	120
	actin-R	TGGCGCGATGATCTTAATTT	
